# Subchronic Toxicities of Four Per- and Polyfluoroalkyl Substances (PFASs) by Oral Exposure in Sprague–Dawley Rats

**DOI:** 10.3390/toxics13070524

**Published:** 2025-06-22

**Authors:** Elaina M. Kenyon, Michael J. Devito, Grace Patlewicz, Linda D. Adams, Russell S. Thomas, Jeffrey L. Ambroso, Xi Yang, James C. Blake, Bindu G. Upadhyay, Johnathan Furr, Michael F. Hughes

**Affiliations:** 1 Center for Computational Toxicology and Exposure, Office of Research and Development, U.S. Environmental Protection Agency, Durham, NC 27709, USA; devito.michael@epa.gov (M.J.D.); patlewicz.grace@epa.gov (G.P.); adams.lindad@epa.gov (L.D.A.); thomas.russell@epa.gov (R.S.T.); hughes.michaelf@epa.gov (M.F.H.); 2 RTI International, Durham, NC 27709, USA; jambroso@rti.org (J.L.A.); xyang@rti.org (X.Y.); jcblake@rti.org (J.C.B.); bupadhyay@rti.org (B.G.U.); 3 Inotiv, Gaithersburg, MD 20878, USA; jfurr@inotiv.com

**Keywords:** Per- and polyfluoroalkyl substances, subchronic oral toxicity, methyl heptafluoropropyl ketone, 1H,1H,2H,2H-perfluorohexyl iodide, 2-chloro-2,3,3,3-tetrafluoropropanoic acid, 1H,1H,9H-perfluorononyl acrylate

## Abstract

PFASs are widely present and persistent in the environment, and exposure can occur via multiple pathways. Human and animal PFAS exposures have been associated with alterations in thyroid hormones, hepatotoxicity, and other adverse effects. This study evaluated the subchronic toxicities of four specific PFASs in 90-day oral rat studies. Studies were conducted in male and female Sprague–Dawley rats exposed to PFASs in corn oil via oral gavage. The PFASs studied were 1H,1H,9H-perfluorononyl acrylate (PFNAC), 1H,1H,2H,2H-perfluorohexyl iodide (PFHI), methyl heptafluoropropyl ketone (MHFPK), and 2-chloro-2,3,3,3-tetrafluoropropanoic acid (CTFPA). High doses were 10 mg/kg-day (male) and 30 mg/kg-day (female) for PFNAC, 200 mg/kg-day for PFHI, 300 mg/kg-day for MHFPK, and 30 (male) and 100 mg/kg-day (female) for CTFPA. The four lower doses for each PFAS were spaced at two- or threefold dose increments. The most consistent effect was dose-dependent increases in the relative and absolute liver weights for PFNAC, PFHI, and CTFPA but not for MHFPK. Increased liver weights were correlated with findings of hepatocellular hypertrophy. Increased kidney weights for PFNAC and PFHI were correlated with increased incidence of minimal tubule epithelial hypertrophy (PFNAC) or increased incidence and severity of chronic progressive nephropathy and hyaline droplet accumulation (PFHI). There were no compound-related effects on morbidity and mortality or overt signs of toxicity.

## 1. Introduction

Per- and polyfluoroalkyl substances (PFASs) are synthetic chemicals used in industrial applications and found in numerous commercial products. The exact scope and scale of the PFAS class are challenging to quantify, in part since there have been many ways to define what constitutes a PFAS [1,2,3]. Several Toxic Substances Control Act (TSCA) activities offer a more focused definition such that PFASs must include one of three substructures: (1) R-(CF2)-CF(R′)R″, where both the CF2 and CF moieties are saturated carbons; (2) R-CF2OCF2-R′, where R and R′ can be F, O, or saturated carbons; or (3) CF3C(CF3)R′R″, where R′ and R″ can either be F or saturated carbons [4]. In either case, the scale of the potential PFASs captured from these definitions comprises several thousand substances at a minimum, the vast majority of which have not been studied.

PFASs, as a large and diverse chemical class, present many toxicologic challenges. PFAS exposure has been associated with human health impacts [5,6]. Most of the effects have been studied with the legacy PFASs, perfluorooctanoic acid (PFOA) and perfluorosulfonic acid (PFOS). Associations between PFAS exposure and adverse health effects have been reported in multiple epidemiologic studies. Effects include changes in immune function (reduced responses to vaccines), altered prenatal development (lower birth weights), lipid and insulin dysregulations, and liver and kidney diseases [5,6]. However, mammalian bioassay and epidemiological data are available for only 41 PFASs [7]. Thus, the vast majority of PFASs in commerce are considered as “data poor” in that there are no publicly available data to inform human health hazard assessments.

Given that there is a large number of PFASs in commerce, it is improbable that toxicological data will be generated for use in human health hazard assessments for each of these unique chemicals. The U.S. Environmental Protection Agency (EPA) has published a national testing strategy that groups PFASs into structurally similar categories that serve as the basis for both identifying PFAS chemicals for testing as well as allowing the EPA to establish toxicity levels for PFASs with the identified categories [4]. In the present study, we evaluated the subchronic oral toxicities of four specific PFASs in 90-day studies in rats to provide additional in vivo mammalian toxicity data for such comparisons. These PFASs were selected to represent prioritized data-poor chemical categories of PFASs, as initially developed in Patlewicz et al. [8]. The studies were conducted in male and female Sprague–Dawley rats exposed to each of the four PFASs in corn oil via oral gavage. The PFASs studied were 1H,1H,9H-perfluorononyl acrylate (PFNAC; CASRN 4180-26-1), 1H,1H,2H,2H-perfluorohexyl iodide (PFHI; CASRN 2043-55-2), methyl heptafluoropropyl ketone (MHFPK; CASRN 355-17-9), and 2-chloro-2,3,3,3-tetrafluoropropanoic acid (CTFPA; CASRN 6189-02-2).

## 2. Materials and Methods

### 2.1. Test Substances

**Figure 1:**
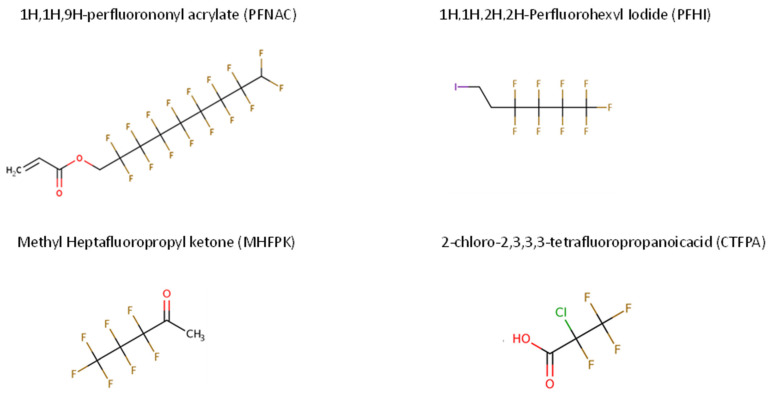
Chemical structures for PFAS compounds studied. Figures are from the EPA’s CompTox Chemicals Dashboard (https://comptox.epa.gov/dashboard/; accessed on 3 June 2025).

Four PFAS chemicals were selected to encompass a range of data-poor PFAS chemical categories and structures lacking in vivo repeated-dose information: 1H,1H,9H-perfluorononyl acrylate (PFNAC), 1H,1H,2H,2H-perfluorohexyl iodide (PFHI), methyl heptafluoropropyl ketone (MHFPK), and 2-chloro-2,3,3,3-tetrafluoropropanoic acid (CTFPA). These categories had been prioritized based on their in vivo data, the ability to procure them in sufficient quantities for 90-day in vivo studies, and the likelihood to pass analytical chemistry detection and be stable in dosing solutions. The chemical identity information is provided in Table 1, and the chemical structures are shown in Figure 1. These PFASs are linear carbon chains, from C3 to C9, with functional groups including an acrylate, a halide, a carboxylic acid, and a ketone. The acidic compound (CTFPA) has one Cl atom in place of an F atom. The chemicals were acquired from SynQuest Laboratories (Alachua, FL, USA) and were >97% (PFNAC) or >99% (PFHI, MHFPK, and CTFPA) pure.

### 2.2. Formulation and Analysis of Dosing Solutions

Based on solubility, corn oil (Welsh, Holme & Clark Inc., Newark, NJ, USA) was used as the vehicle for all four PFASs. Gas chromatography (GC) with flame ionization detection (FID) was used for the analysis of the PFNAC, PFHI, and MHFPK formulations, and a high-performance liquid chromatography (HPLC) method with ultraviolet spectroscopy was used for the CTFPA formulation. The details of the dosing solution analytical conditions are summarized in supplemental Tables S1 and S2. These analytical methods were developed specifically for these subchronic toxicity studies. Stability studies demonstrated that all four PFASs are stable for at least 42 days in the corn oil vehicle. Dosing solutions were prepared and replaced between at least every 29 days and no more than every 39 days.

### 2.3. Animals

Male and female Sprague–Dawley rats (Charles River Breeding Labs, Raleigh, NC, USA) 8–9 weeks old and weighing a minimum of 150 g at the initiation of the dosing, were used in these studies. The rats were acclimated to the facility for at least 5 days prior to dosing. Up to 3 rats were housed in polycarbonate cages with hardwood bedding (Teklad SaniChip, certified 7090, Inotiv, Lafayette, IN, USA) and fed a pelleted diet (Certified Global Teklad Laboratory 2018 Diet, Inotiv, Lafayette, IN, USA) ad libitum while receiving filtered water via an automatic watering system and/or water bottles. The room’s environmental conditions were from 20 to 26 °C and from 30 to 70% humidity, with a 12/12 h light/dark cycle and a minimum of 10 air changes per hour. All the study protocols were reviewed and approved by the contractor, Institutional Animal Care and Use Committee, in accordance with the provisions of the Animal Welfare Act, PHS Policy on Humane Care and Use of Laboratory Animals, and U.S. Interagency Research Animal Committee Principles for the Utilization and Care of Research Animals and were conducted in an AAALAC-accredited facility. The rats were euthanized using carbon dioxide followed by bilateral thoracotomy.

### 2.4. Study Design

Tolerability, 14-day dose-range-finding, and 90-day subchronic toxicity studies were conducted at Inotiv (Gaithersburg, MD, USA), monitored by the Research Triangle Institute (RTI) (Durham, NC, USA) and sponsored and funded by the EPA. Tolerability studies used four PFAS dose levels plus a control, with the high doses not to exceed 1000 mg/kg (subject to solubility in corn oil, dose volume of 5 or 10 mL/kg) and lower doses separated by threefold spacing. The dose levels for PFHI, MHFPK, and CTFPA were 0, 30, 100, and 1000 mg/kg. The dose levels for PFNAC were 0, 25, 75, 225, and 667 mg/kg. Tolerability was studied using male rats (*n* = 3 rats/group) that received a single dose by oral gavage. The rats were euthanized at 48 hours post dosing. The data collected were bodyweight (BW) and BW changes (BWCs), feed consumption (FC) and water consumption (WC), cage-side observations, and physical exams; no necropsies were performed.

Dose-range-finding (14-day) studies were conducted using male and female rats to establish dose levels for 90-day studies and identify potential target organ toxicities. Male and female rats (*n* = 5 sex/group, a control and three dose groups, corn oil vehicle dose volume of 5 mL/kg) were administered PFASs by oral gavage daily for 14 days and euthanized on study day (SD) 15. The dose levels for PFHI, CTFPA, and MHFPK were 0, 30, 100, and 300 mg/kg-day. The dose levels for PFNAC were 0, 10, 30, and 100 mg/kg-day. The in vivo data collected included BW, BWCs, FC, cage-side observations, and physical exams. Gross necropsies, relative and absolute organ weights, and clinical chemistry data were collected for all the groups. Macroscopic and microscopic pathology examinations were conducted for the control and high-dose groups initially; the other groups were examined if justified by experimental findings. Plasma samples were analyzed ~2 h post dosing on day 1 and at day 15 for the parent PFAS chemical.

Subchronic (90-day) studies were performed in accordance with current U.S. EPA’s Toxic Substances Control Act Good Laboratory Practice (GLP) Standards [9], with the following two general exceptions to the GLP guidelines: Analysis of the dose formulations was conducted according to RTI-approved Standard Operating Procedures, and corn oil (the vehicle) was not characterized (certificate of analysis from the vendor is available in the appendices to the final reports). Male and female Sprague–Dawley rats (five dose levels plus a control, *n* = 10/sex/dose group, corn oil vehicle dose volume of 3 mL/kg; oral gavage) were used in these studies, and the rats were euthanized on SD 91. The dose levels for PFHI were 0, 12.5, 25, 50, 100, and 200 mg/kg-day. The dose levels for MHFPK were 0, 18.8, 37.5, 75, 150, and 300 mg/kg-day. The dose levels for PFNAC were 0, 0.1, 0.3, 1.0, 3.0, and 10 mg/kg-day for male rats and 0, 0.3, 1.0, 3.0, 10, and 30 mg/kg-day for female rats. For CTFPA, the dose levels were 0, 1.9, 3.8. 7.5, 15, and 30 mg/kg-day for male rats and 0, 6.3, 12.5, 25, 50, and 100 mg/kg-day for female rats. For each PFAS, three batches of dosing solution were prepared. The time between the batch preparation of the dosing solutions ranged between 29 and 39 days. Blood samples were collected from all the rats at termination for serum chemistry, hematology, coagulation, and the analysis of the parent PFAS chemical in the plasma. In vivo data collection and further detail on the endpoints examined postmortem are described below.

#### 2.4.1. In Vivo Observations

The rats were observed twice daily for morbidity and mortality throughout the study period. At least once prior to randomization to test groups and weekly thereafter, the rats were physically examined, and bodyweights were obtained. Feed consumption was quantified weekly starting on SD 1.

Neurotoxicity was evaluated using the functional observational battery (FOB) and measuring locomotor activity (LMA) 2–4 h post dosing on SDs 78–80 and 80–84, respectively. For both the 14- and 90-day studies, an ophthalmologic examination was conducted prior to randomization and near the end of the study, using indirect ophthalmoscopy and slit–lamp biomicroscopy (as needed) after the administration of a mydriatic solution.

#### 2.4.2. Hematology and Serum Chemistry

Hematology, biochemistry, and coagulation endpoints were measured at the termination of the study. A Siemens Advia 2120i hematology analyzer (Siemens Healthineers, Erlangen, Germany) was used to measure (or calculate) the parameters related to the complete blood count, leukocyte differential count, and reticulocyte count. The hematology parameters are listed and described in Table 2. A Siemens Dimension Xpand chemistry analyzer was used to measure the serum clinical chemistry parameters listed in Table 3. The prothrombin time (PTT) and activated partial thromboplastin time (APTT) were measured using an Instrumentation Laboratory (Bedford, MA, USA) ACL Elite Pro coagulation analyzer (laser–nephelometric centrifugal method).

#### 2.4.3. Postmortem Evaluations

At necropsy, the rats were visually examined for external abnormalities and organ weights (absolute and relative to the BW and brain weight), which were recorded (paired organs weighed together). The tissues were preserved in 10% neutral buffered formalin (NBF), except for the ocular tissues, testes, and epididymides, which were preserved in modified Davidson’s fixative and later transferred to 10% NBF. Bone marrow smears were prepared from the femurs, and the slides were air-dried, fixed in methanol, stained, and stored at room temperature for possible future evaluation. The preserved tissues were embedded in paraffin, sectioned, stained with hematoxylin and eosin, and examined by a board-certified veterinary pathologist.

#### 2.4.4. PFAS Plasma Sampling and Analysis

On SD 1 of the 14-day study (~2 h post dosing), 0.2 mL of blood was collected via tail vein puncture in tubes containing K3EDTA and stored on wet ice. Prior to the scheduled euthanasia, blood (~1 mL) was collected from the jugular vein in tubes containing K3EDTA and stored on wet ice on SD 15, for the 14-day study, and SD 91, for the 90-day study. All the tubes were centrifuged at an ~1500 relative centrifugal force within 1 h of collection for 15 min at 5 ± 3 °C. The plasma was stored at −75 ± 15 °C until the analysis. PFNAC and PFHI were analyzed using a headspace GC–mass spectrometry (MS) method, as summarized in Table S3, and CTFPA was analyzed using an ultra-HPLC-MS method, as summarized in Table S4. The MHFPK samples were not analyzed because a method suitable for plasma was not available. The details concerning the preparation of the samples, method blanks, and matrix and QC standards for the analysis are available in the appendices to the study reports.

### 2.5. Statistical Analysis

Statistical analysis was performed using Provantis™ Version 10 or greater (Instem LSS, Limited; Stone, Staffordshire, UK). Differences among the treatment groups compared to the control were evaluated using one-way analysis of variance (ANOVA) if the underlying assumptions of a normal distribution (Shapiro–Wilk test) and the homogeneity of the variance (Levene’s test) were satisfied. If the overall ANOVA was significant, Dunnett’s *t*-test was used to determine which groups differed from the controls. Significance was set at the 0.05 level (two-sided). If the normality or homogeneity of the variance assumptions was not met (*p* ≤ 0.01), the data were log10-transformed, and Shapiro–Wilk and Levene’s tests were repeated. If the assumptions of normality and homogenous variance among the groups were met, these log10-transformed data were used for statistical analysis. If either or both conditions of normality and the homogeneity of the variance was/were not met using the log10-transformed data, the data were rank-transformed, and the nonparametric Kruskal–Wallis ANOVA was used. Benchmark dose (BMD) analysis was performed using excel-based benchmark dose (BMD) software version 3.3.2 [10] and the methods described by the U.S. EPA [11,12].

## 3. Results

### 3.1. Dose Formulation and Stability

PFNAC, PFHI, and MHFPK were soluble in corn oil up to 67, 200, and 100 mg/mL, respectively. As solubility in aqueous vehicles was 40 mg/mL or less for all three chemicals, corn oil was chosen as the vehicle. PFNAC was stable at the ambient temperature for 51 days at target concentrations of 0.2 and 10 mg/mL PFHI was stable in corn oil at ambient temperatures and target concentrations of 1 and 80 mg/mL for up to 70 days. MFHPK was stable in corn oil at room temperature for up to 44 days at target concentrations of 5 and 100 mg/mL. CTFPA was soluble in both deionized (DI) water and corn oil at 200 mg/mL when stored refrigerated overnight. However, the pH of the DI water solution was <1, and attempts to raise the pH resulted in the precipitation of the CTFPA. Thus, corn oil was chosen as the vehicle for CTFPA. The stability of the CTFPA in the corn oil was evaluated for 8, 13, 15, 23, 35, and 42 days stored refrigerated in plastic bottles. CTFPA at 40 mg/mL was stable for up to 42 days. CTFPA at 0.6 mg/mL was stable for up to 22 days when refrigerated in plastic bottles but by 42 days had decreased to approximately 81%.

### 3.2. Tolerability and Dose Range Finding Studies

No compound-related morbidity, mortality, or overt toxic effects were observed in tolerability studies. The results for these studies are summarized in Table 4. Based on the results from the tolerability studies, high doses of 100 mg/kg-day (PFNAC) and 300 mg/kg-day (PFHI, MFHPK, and CTFPA) were chosen for 14-day dose-range-finding studies with three-fold spacing of lower doses. Among all four PFASs studied, there were no substance-related effects on mortality, clinical observations, bodyweight changes, ocular lesions, coagulation endpoints, or gross lesions observed at necropsy in the 14-day studies. The key substance-related findings from these studies are summarized in Table 5. The liver was identified as a potential target organ for PFNAC, PFHI, and CTFPA on the basis of increased relative and absolute liver weights and hepatocellular hypertrophy.

### 3.3. Subchronic Studies

Based on the results of the 14-day studies, high doses of 10 and 30 mg/kg-day for PFNAC and 30 and 100 mg/kg-day for CTFPA were selected for males and females, respectively. In the case of the PFHI and MHFPK, high doses of 200 and 300 mg/kg-day, respectively, were selected for both sexes. Descriptive results for the 90-day studies are summarized in Table 6. There were no compound-related effects on morbidity, mortality, or overt signs of toxicity. Additionally, there were no PFAS-related ocular lesions or changes in locomotor activity or findings in the functional observational battery evaluating potential neurological changes. Changes in bodyweight, bodyweight gain, and feed consumption are described qualitatively in Table 6 and numerically summarized in Tables S5 and S6 for male and female rats, respectively. More detailed data are available in the individual study reports and their appendices. In general, bodyweight changes and feed consumption were not affected by PFAS exposure, or the effects did not display dose-dependent, time-dependent, or interrelated trends. As an example of the latter for CTPFA, dose-dependent decreases in feed consumption in male rats at > 3.8 mg/kg-day were not accompanied by changes in bodyweight gain.

**Figure 2:**
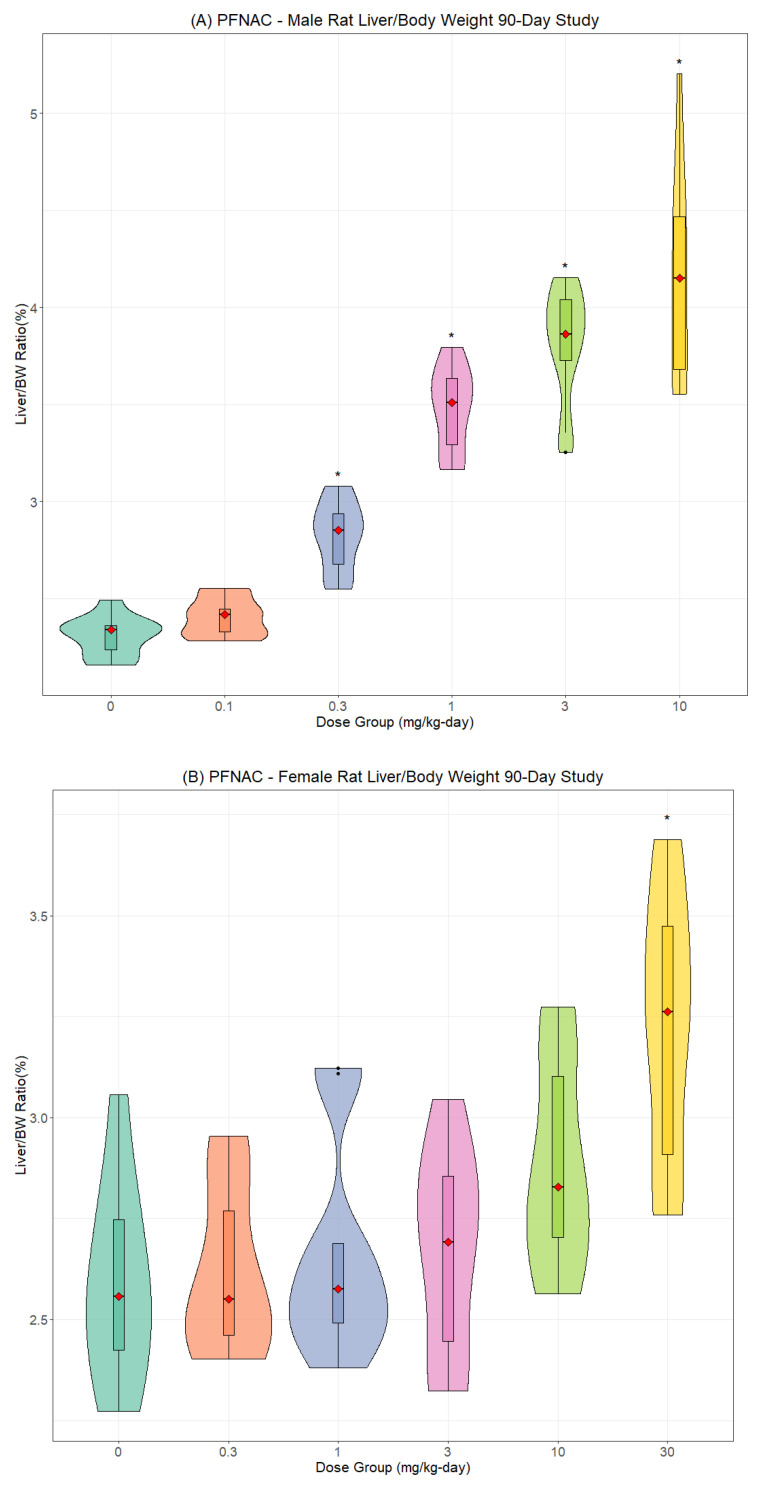
Relative liver weights for male (**A**) and female (**B**) SD rats exposed to PFNAC by oral gavage in corn oil for 90 days (n = 10/sex/dose). Male data were analyzed using Kruskal–Wallis ANOVA and Dunnett’s test based on ranks. Female data were analyzed using ANOVA and Dunnett’s test. The data are displayed as violin plots, which are a combination of a box plot and a probability density function (PDF). The box plot shows the median (red diamond), interquartile range (box), and range of the data (horizontal bars). The PDF illustrates the distribution or shape of the data set; * indicates statistically different from the control (*p* < 0.05).

**Figure 3:**
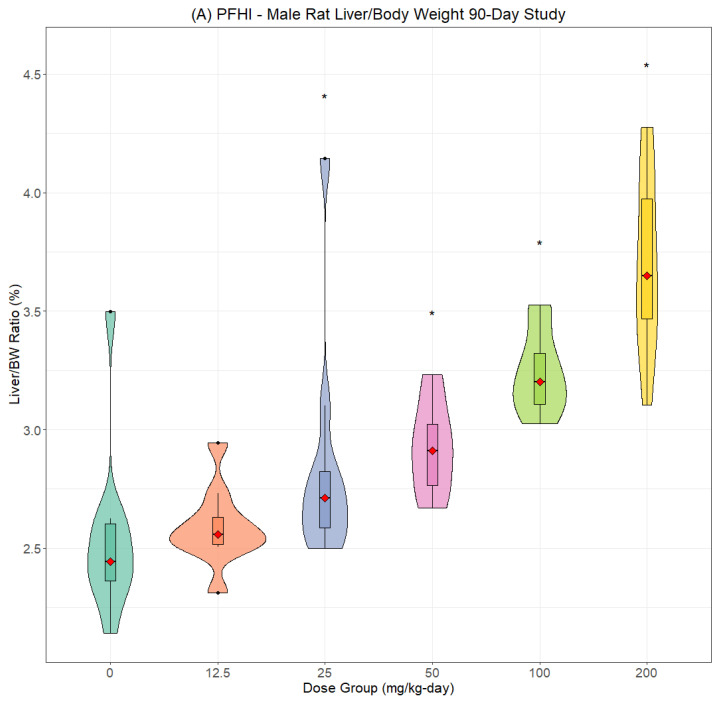

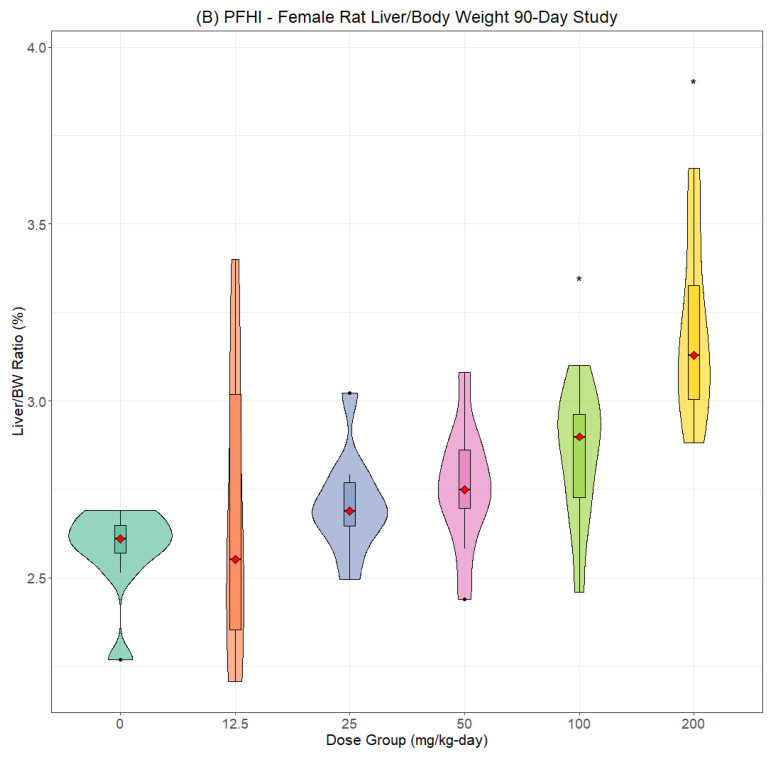
Relative liver weights for male (**A**) and female (**B**) SD rats exposed to PFHI by oral gavage in corn oil for 90 days (n = 10/sex/dose). Male and female data were analyzed using Kruskal–Wallis ANOVA and Dunnett’s test based on ranks. The data are displayed as violin plots, which are a combination of a box plot and a probability density function (PDF). The box plot shows the median (red diamond), interquartile range (box), and range of the data (horizontal bars). The PDF illustrates the distribution or shape of the data set; * indicates statistically different from the control (*p* < 0.05).

**Figure 4:**
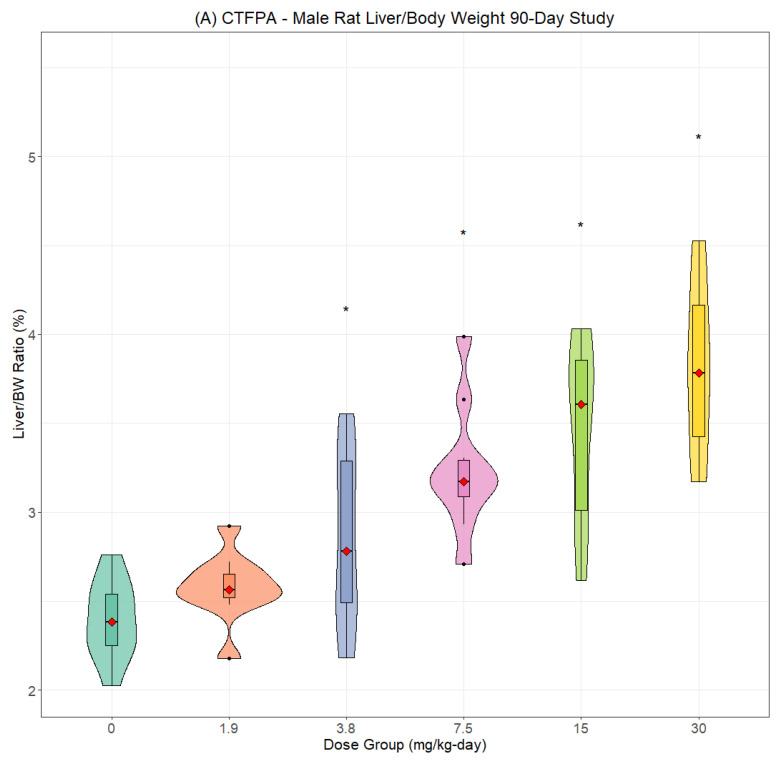

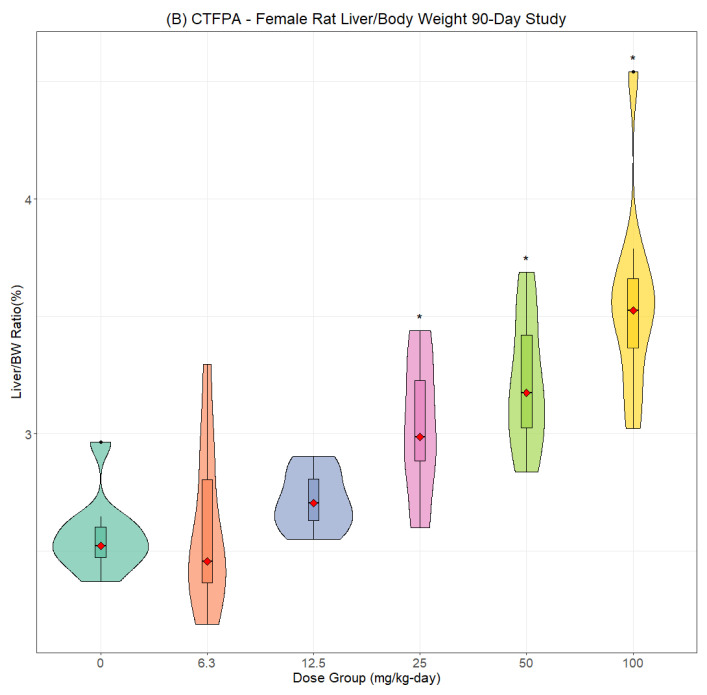
Relative liver weights for male (**A**) and female (**B**) SD rats exposed to CTFPA by oral gave in corn oil for 90 days (n = 10/sex/dose). Male data were log-transformed and analyzed using ANOVA and Dunnett’s test. Female data were analyzed using ANOVA and Dunnett’s test. The data are displayed as violin plots, which are a combination of a box plot and a probability density function (PDF). The box plot shows the median (red diamond), interquartile range (box), and range of the data (horizontal bars), The PDF illustrates the distribution or shape of the data set; * indicates statistically different from the control (*p* < 0.05).

**Figure 5:**
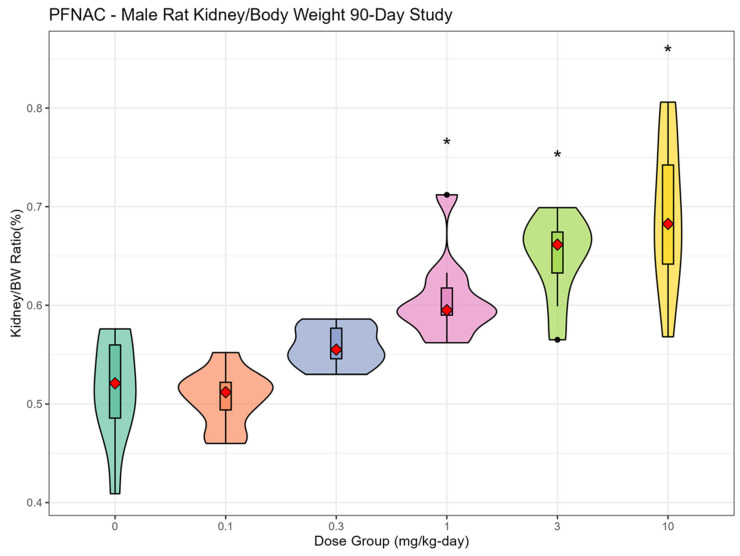
Relative kidney weights for male SD rats exposed to PFNAC by oral gavage in corn oil for 90 days (n = 10/dose). Male and female data were analyzed using ANOVA and Dunnett’s test. The data are displayed as violin plots, which are a combination of a box plot and a probability density function (PDF). The box plot shows the median (red diamond), interquartile range (box), and range of the data (horizontal bars). The PDF illustrates the distribution or shape of the data set; * indicates statistically different from the control (*p* < 0.05).

**Figure 6:**
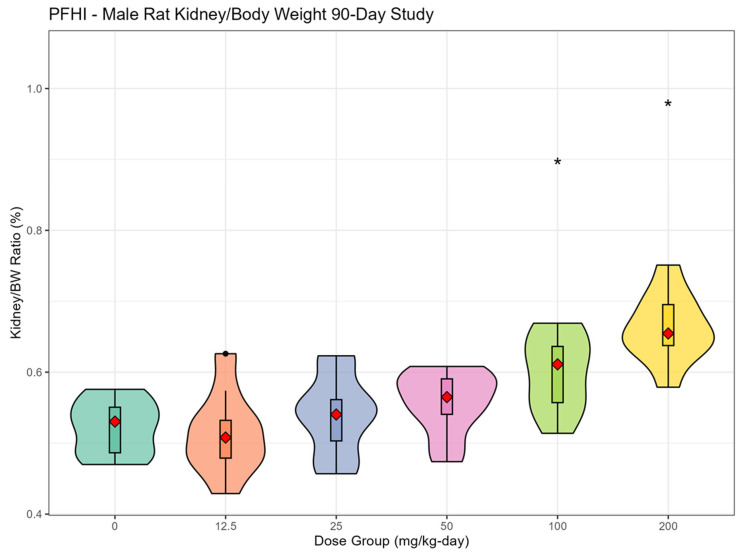
Relative kidney weights for male SD rats exposed to PFHI by oral gavage in corn oil for 90 days (n = 10/sex/dose). Data were analyzed using ANOVA and Dunnett’s test. The data are displayed as violin plots, which are a combination of a box plot and a probability density function (PDF). The box plot shows the median (red diamond), interquartile range (box), and range of the data (horizontal bars), The PDF illustrates the distribution or shape of the data set; * indicates statistically different from the control (*p* < 0.05).

The most consistent compound-related effect was dose-dependent increases in the relative and absolute liver weights for PFNAC (Figure 2A,B), PFHI (Figure 3A,B), and CTFPA (Figure 4A,B) but not for MHFPK (Tables S5–S8). Increased liver weights were correlated with increased incidence of histological findings of hepatocellular hypertrophy. Liver weight data for all four PFASs are summarized in Table S5. No-observed effect levels (NOELs), the lowest-observed effect levels (LOELs), and BMD results are summarized in Table 7. Male rats were consistently more sensitive to liver weight changes compared to female rats. Liver weight changes occurred at lower doses in male rats compared to female rats, and the magnitude of the liver weight changes was greater for male compared to female rats. Another common finding was increased relative and absolute kidney weights for PFNAC (Figure 5) and PFHI (Figure 6), which were correlated with minimal tubule epithelial hypertrophy (PFNAC) or increased incidence and severity of chronic progressive nephropathy and hyaline droplet accumulation (PFHI). Kidney weight data for all four PFASs are summarized in Table S8. NOEL, LOEL, and BMD results are summarized in Table 7. Male rats were also more sensitive to kidney weight changes compared to female rats. However, changes in relative and absolute kidney weights were milder compared to liver weight changes.

No other dose-dependent organ (i.e., non-hepatic) weight changes were observed for PFNAC and PFHI. However, for CTFPA, both absolute and relative thymus weights were significantly increased at the highest dose (30 mg/kg-day) in males but not in females (at the highest dose of 100 mg/kg-day). For MHFPK, in male rats, there was a significant decrease (20%) in the adrenal/BW ratio at 150 mg/kg-day but no effect at 300 mg/kg-day in male rats. No effects of MHFPK on organ weights in female rats were observed.

Changes in coagulation parameters were observed across all four PFASs (Table S9). For PFNAC, in male rats only, there were mild shortened APTTs at 1 mg/kg-day. While statistically significant, these changes are not considered as biologically significant and were possibly an indirect effect related to reduced BW gains observed in males at 3 and 10 mg/kg-day. Both male (≥12.5 mg/kg-day) and female (≥25 mg/kg-day) rats exposed to PFHI exhibited from minimal to mild prolongations in prothrombin times (PTTs). From minimal to mild dose-dependent shortened APTT was observed only in male rats administered ≥3.8 mg/kg-day of CTFPA. Also, in males only, there was a generally dose-dependent minimal-to-mild increase in PTT at ≥37.5 mg/kg-day in rats administered MFHPK.

Various hematology parameters were altered across the studied PFASs for both the male and female rats, although males tended to be more sensitive. Selected hematology data for some of the more commonly affected hematologic parameters are shown in Tables S10 and S11 for male and female rats, respectively. Further detail is available in the study reports and associated data tables. For PFNAC, significant alterations were limited to males for hemoglobin (HGB), hematocrit (HCT), erythrocyte count (RBC), absolute numbers of reticulocytes (ABRETis), neutrophils, and eosinophils. More hematologic parameters were altered for PFNAC-exposed rats compared to rats exposed to the other PFASs. Male rats, but not female rats, exposed to CTFPA displayed increases in RBC, HCT, and HGB, indicative of increased red cell mass. In the case of PFHI, minimal decreases in ABRETis were observed in male but not in female rats exposed at ≥50 mg/kg-day. Changes in the mean platelet volume (MPV) occurred mainly in female rats administered 200 mg/kg-day of PFHI, and sporadic increases in the MPV (not statistically significant but likely PFHI related) also occurred in male and female rats exposed to ≥50 mg/kg-day of PFHI. Both sexes exposed to ≥18.8 mg/kg-day of MHFPK showed from minimal to mild dose-dependent increases in the MPV. Other fluctuations in the mean and individual values for hematologic parameters were not considered as test substance related because they occurred sporadically or were negligible in magnitude and consistent with biologic and/or analytical variation(s).

Alterations in the more commonly affected clinical chemistry parameters for the studied PFASs are shown in Tables S12 and S13 for male and female rats, respectively. Further detail is available in the study reports and associated data tables. For PFNAC, there were mild decreases in cholesterol and mild increases in glucose at ≥0.1 mg/kg-day in male rats and at 30 mg/kg-day in female rats, suggesting altered energy metabolism. Mild increases in alkaline phosphatase (ALPi) were observed in males only at ≥0.3 mg/kg-day, and globulin was from minimally to mildly decreased in males only at ≥1.0 mg/kg-day. Changes in albumin (ALB) and globulin (GLOB) together resulted in increased albumin/globulin (A/G) ratios. Other alterations observed in clinical chemistry parameters in PFNAC-dosed rats occurred sporadically and were likely not directly attributable to PFNAC exposure. For PFHI, there were from minimal to mild dose-dependent increases in cholesterol in males at ≥25 mg/kg-day and in females at 200 mg/kg-day. There were PFHI-related mild increases in the total protein, resulting from increases in both albumin and globulin, in females at 200 mg/kg/day. Male rats at all the dose levels had mild decreases in calcium, phosphorus, potassium, and/or creatinine kinase (CK). These findings did not follow a dose-dependent trend and were likely indirect effects related to decreases in bodyweight gains. In the case of the CTFPA, from minimal to mild increases in albumin and the A/G ratio occurred in male rats at ≥7.5 mg/kg-day and in female rats at ≥50 mg/kg-day. The findings observed only in the male CTFPA-dosed rats included from minimal to mild decreases in globulin at ≥7.5 mg/kg-day, from minimal to mild increases in ALPi at ≥7.5 mg/kg-day, and minimally increased BUN at 30 mg/kg-day. For MFHPK, the fluctuations in the clinical chemistry parameters tended to be inconsistent across the dose groups (lack of a dose response) and varied between individuals (increased or decreased), rendering the relationship of these changes to MFHPK as uncertain.

### 3.4. Rat Plasma Bioanalytical Results

The bioanalytical results for the plasma in the 14-day studies are summarized in Table 8 for PFNAC, PFHI, and CTFPA. For PFNAC, the parent chemical was not detected in any representative samples analyzed (at the highest dose level) at 1 (~2 h post dosing) or at 15 days, suggesting very rapid metabolism; a single large peak was detected that is likely associated with a metabolite. In the case of the PFHI, there were dose-dependent increases in the plasma concentration, with no clear difference between the sexes; an approximately 10- to 100-fold lower plasma concentration for SD 15 compared to SD 1 suggests rapid clearance with no accumulation. For CTFPA, there were no major differences between the sexes, and there were dose-dependent increases in the plasma concentration with large within-group variation. CTFPA exhibits relatively slow plasma clearance compared to those of the other PFASs evaluated, without clear evidence of parent chemical accumulation in the plasma during the 14-day study period.

**Figure 7:**
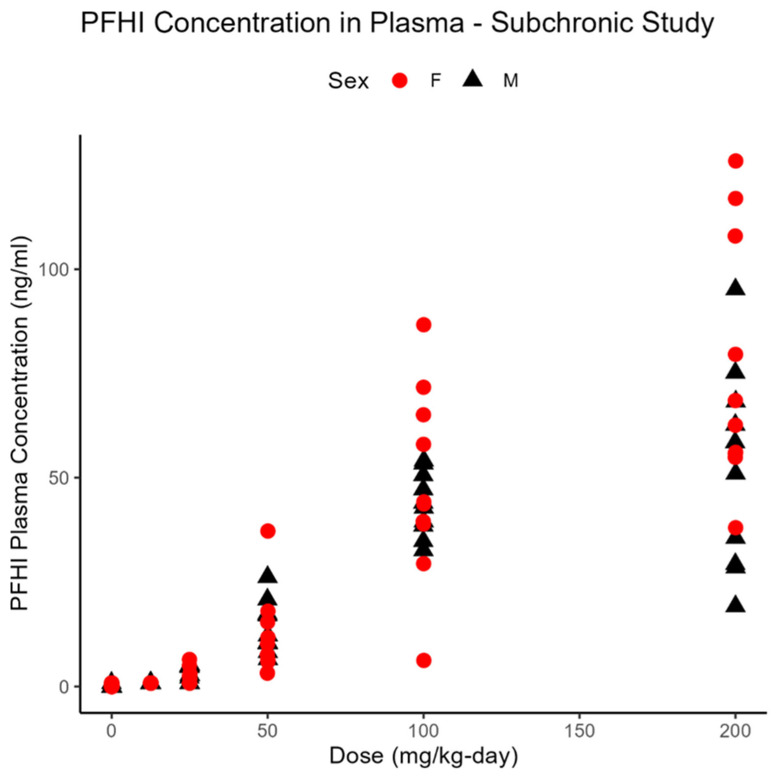
PFHI concentrations in the plasma of individual male and female SD rats (n = 10/sex/dose) after 90 days of subchronic exposure to PFHI by oral gavage in corn oil. Samples were taken on Day 91 and analyzed using GC-MS.

**Figure 8:**
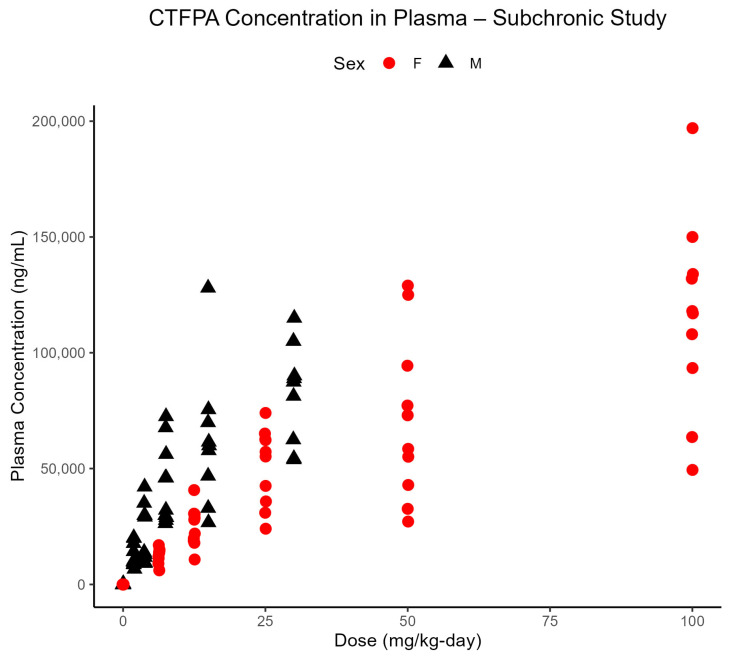
CTFPA concentrations in the plasma of individual male and female SD rats (n = 10/sex/dose) after 90 days of subchronic exposure to CTFPA by oral gavage in corn oil. Samples were taken on Day 91 and analyzed using ultra-HPLC-MS. Note that the doses differed for the males and females, which accounts for the symbols being offset from each other.

The bioanalytical results for the plasma in the 90-day studies are summarized in Table 9. Consistent with results from the 14-day studies, PFNAC was not detected in the plasma, but a large peak that is likely associated with a metabolite was observed. The PFHI plasma concentration increased in a dose-dependent manner, with no consistent sex-dependent differences (Figure 7). Considered in the context of the results from the 14-day studies, PFHI demonstrated rapid clearance without significant accumulation in the plasma. In the case of the CTFPA, there is evidence of accumulation in the plasma when the final plasma concentrations are compared for the 14- and 90-day studies (Figure 8) at the same dose level. In male rats at 30 mg/kg-day, the percentage of the dose in the plasma is 6.39 ± 2.03 (mean ± standard deviation) vs. 10.82 ± 2.76 at 14 and 90 days, respectively. In female rats at 100 mg/kg-day, the percentage of the dose in the plasma is 1.75 ± 0.67 vs. 4.48 ± 1.63 at 14 and 90 days, respectively.

## 4. Discussion

The most common finding among the PFASs evaluated in these subchronic rat studies was increased relative and absolute liver weights, which were associated with both increased incidence and severity of hepatocellular hypertrophy. PFNAC exhibited the most severe effects, followed by CFTPA and PFHI, with no statistically significant effects on the absolute or relative liver weights observed for MHFPK. The male rats were more sensitive to hepatic effects compared to the female rats. Increased relative and absolute kidney weights for PFNAC and PFHI were correlated with minimal tubular epithelial hypertrophy (PFNAC) or increased incidence and severity of chronic progressive nephropathy and hyaline droplet accumulation (PFHI). The male rats were also more sensitive to renal effects compared to the female rats. Similar hepatic and renal changes have previously been reported in rats exposed to various PFASs, with males being more generally sensitive compared to female rats (e.g., the National Toxicology Program (NTP)) [13,14,15].

The hepatic changes observed following PFAS administration in our studies are not specifically adverse. The effect levels in Table 7 are designated as no effect or the lowest effect levels rather than adverse effect levels because these effects can be considered as potentially adaptive and reversible. This is because of the absence of pathological changes in the liver and the lack of consistent adverse effects on biomarkers indicative of liver injury. The effects on the liver by the PFASs observed in our studies are potentially reversible based on studies of hepatic injury in general. For example, Butenoff et al. [16] reported that the liver weights of male rats administered ammonium perfluorobutyrate by oral gavage, which had increased following 28- and 90-day exposures, returned to control levels following a 3-week recovery period after the completion of the dosing regimen. There were no effects of ammonium perfluorobutyrate on the liver weight in female rats. The hepatocellular hypertrophy in male rats in the 28- and 90-day exposures were also resolved by the end of the recovery period. Amacher et al. [17] reported that although liver weight increases of 20% or greater are associated with hepatic hypertrophy and cytochrome P450 enzyme induction, the extent of the hypertrophy and magnitude of the liver weight changes were neither correlated nor associated with elevated serum enzymes indicative of liver injury. It is worth noting that longer durations of exposure can potentially lead to liver toxicity [18].

The liver has been reported to be a sensitive organ to PFAS-induced toxicity in mammals [19,20]. A part of this effect is due to the liver being the first organ a systemically absorbed PFAS would contact following oral exposure. Three of the four PFAS compounds tested in the present study increased the liver weight, and the affected livers displayed hepatomegaly. In NTP 28-day toxicity studies of PFOA, PFOS, perfluorohexanoic acid, perfluorononanoic acid, perfluorodecanoic acid, perfluorobutane sulfonic acid (PFBS), and perfluorohexane sulfonate potassium salt (PFHxSK+), common findings were increased liver and kidney weights, hepatocyte hypertrophy, increased hepatocyte cytoplasmic alterations, and decreased serum cholesterol, serum thyroxine, and triiodothyronine [14,15]. All the PFAS carboxylates and sulfonates tested by NTP increased the absolute and relative weights of the livers of the treated animals. For the four carboxylates, microscopic examination of the livers of the treated male and female rats showed hepatocyte hypertrophy and cytoplasmic alterations. Male rats were more sensitive than female rats to the perfluoroalkyl carboxylates. For the three sulfonates, hepatic hypertrophy was reported for PFBS and PFOS in male and female rats, but for PFHxSK+, this effect was only observed in male rats. Hepatocyte alterations were observed in male and female rats administered PFBS and in female rats exposed to PFOS. The effects of PFASs on the livers of rats occur rather quickly, as changes in liver weights were observed in the 14-day study with PFHI, PFNAC, and CTFPA. This is typical of chemicals that cause hepatocellular hypertrophy in rodents. In the case of the PFNAC in the 90-day studies, alterations in glucose, ALPi, BUN, albumin, and globulin correlated with, and were likely related to, increased organ weights and microscopic findings in the liver and kidney. Butenoff et al. [16] reported significant increases in the absolute and relative liver weights only in male rats after 28- and 90-day oral gavage studies with ammonium perfluorobutyrate. No effects on the liver weight were observed in female rats in the same study. However, perfluorobutane sulfonate did not affect the liver weights or bodyweights in male or female rats at doses of up to 600 mg/kg-day following a 90-day oral gavage study [21]. Changes in liver function observed in animal studies have human relevance because alterations in biomarkers of hepatic dysfunction have been reported in human epidemiologic studies [20]. Mezencev et al. [22] recently reported that sex differences in the liver weight responses of rats were not significantly different across over 200 non-PFAS chemicals used in subchronic studies and found in the EPA’s Toxicity Reference Database.

The kidneys were also affected by exposures to PFNAC, PFHI, and CTFPA, with increases in the relative and absolute weights. A stronger case for adversity can be made for the renal compared to the liver effects observed with PFNAC and PFHI because kidney weight changes were accompanied by histopathological lesions, although the severity of the lesions was generally from minimal to mild. Craig et al. [23] found that increases in the absolute kidney weight, although not the relative kidney weight, were predictive of histopathological lesions. In the NTP 28-day studies with four carboxylates, including PFOA, increases in the relative kidney weight were observed in male and female rats. The effects on the absolute weight were mixed, with no effect, decreases, and increases. For the three sulfonates studied by NTP, PFOS did not affect the relative or absolute weight of the kidney in males or females. For the other two sulfonates, there was either no effect or an increase in weight. In two 90-day studies in rats with perfluorobutane sulfonate [21] and ammonium perfluorobutyrate [16], the kidney weights were not affected in male or female rats.

Our results show that as a general trend, male rats are more sensitive to the effects of PFASs than female rats. One potential explanation is sex differences in the pharmacokinetics of PFASs. Kim et al. [24] reported on several pharmacokinetic parameters in male and female rats following oral and intravenous administrations of PFOA, perfluorohexane sulfonic acid (PFHxS) and PFOS. The plasma half-life, time to the maximal plasma concentration after the oral administration (T_max_), and the area under the curve (AUC) of PFOA and PFHxS were significantly lower for female than for male rats, while the clearance and renal clearance (measured after the intravenous administration) were greater for female rats. The clearances of the PFOA and PFHxS were significantly greater in female rats. The plasma concentration time profiles showed large distinctions between male and female rats for PFOA and PFHxS following oral and intravenous administrations [25]. The renal clearances of the PFOA and PFHxS following the intravenous administration were significantly greater for female rats. The renal transport of PFOA is strongly influenced by sex hormones in the rat [25,26,27]. For example, the urinary elimination of PFOA was increased in castrated male rats. The treatment of castrated rats with testosterone decreased the urinary elimination of PFOA, showing the importance of sex hormones in the renal elimination of PFOA [26]. For PFOS, the plasma half-life, T_max_, AUC, clearance, and renal clearance were similar between male and female rats. Thus, sex differences in pharmacokinetics do not apply to every PFAS but may explain the differences in the toxicity observed in the present study. Certainly, additional research is needed to clarify the sex difference observed.

More than 14,000 PFAS chemicals are listed in the EPA’s Comptox Chemicals Dashboard (https://comptox.epa.gov/dashboard/chemical-lists/PFASSTRUCT, accessed on 3 June 2025). Data from the National Health and Nutrition Examination Survey (NHANES, which measures 17 PFAS chemicals in the blood) suggest that 96% or more of the U.S. population has measurable levels of PFASs in their blood, although levels of some legacy PFASs (e.g., PFOA and PFOS) have declined rapidly since U.S. production halted in the early 2000s [28]. Trudel et al. [29] estimated the human exposure to PFOS and PFOA from environmental sources and those related to consumer products. The ranges of estimated human daily non-occupational exposures in North America and Europe were 1–220 ng/kg-day and 1–130 ng/kg-day for PFOS and PFOA, respectively. The lowest dose we used of the four PFASs studied was 0.1 mg/kg-day for CTFPA, which is approximately 450 times greater than highest estimated human daily intake of PFOS. More recently, East et al. [30] estimated human adult daily intakes of 1 and 0.6 ng/kg-day (assuming an adult weight is 70 kg) of PFOA and PFOS, respectively. Thus, the doses used in our study are most likely not representative of human non-occupational exposure to PFASs. However, higher doses were needed in this study, as its objective was to assess the potential toxicity from repeated exposure to a PFAS. The tolerability and 14-day studies aided us in determining what doses the animals could tolerate over a 90-day exposure period.

The widespread use of PFASs in numerous products and applications, as well as their resistance to degradation, bioaccumulative potential, and association with human health effects have made this chemical class a focus for research. However, the large number and structural diversity of PFAS chemicals has posed significant challenges in understanding human health risks. To address these challenges, the EPA has grouped the landscape of PFAS chemicals into structurally similar categories [4,8,31]. These categories are intended to serve as the basis for both identifying PFAS chemicals for testing as well as allowing the EPA to establish toxicity levels for PFASs within the identified categories. The four substances in this study were selected from PFAS categories with limited human health toxicity data. The diversity of the chemical structures among tested PFASs in this study contributes to a more robust database for evaluating potential human health risks.

## 5. Conclusions

The most consistent PFAS-related effect was the dose-dependent increases in the relative and absolute liver weights for PFNAC, PHFI, and CTFPA but not for MHFPK. Increased liver weights were histologically correlated with hepatocellular hypertrophy. The oral dose potency for hepatic effects was PFNAC > CTFPA > PFHI, and male rats were more sensitive than female rats. The liver was the most common target organ. Further study is needed to correlate these in vivo findings with in vitro endpoints to build robust models for class-based toxicity prediction. Such models can improve our ability to prioritize PFASs for more detailed evaluation.

## Figures and Tables

**Table 1:** Chemical identity information.

PFAS Chemical	DTXSID ^1^	CASRN ^2^	Formula	MW ^3^
1H,1H,9H-Perfluorononyl acrylate (PFNAC)	DTXSID00194615	4180-26-1	C_12_H_6_F_16_O_2_	486.152
1H,1H,2H,2H-Perfluorohexyl iodide (PFHI)	DTXSID1047578	2043-55-2	C_6_H_4_F_9_I	373.988
Methyl heptafluoropropyl ketone (MHFPK)	DTXSID00188993	355-17-9	C_5_H_3_F_7_O	212.07
2-Chloro-2,3,3,3-tetrafluoropropanoic acid (CTFPA)	DTXSID30476698	6189-02-2	C_3_HClF_4_O_2_	180.48

^1^ Distributed Structure–Searchable Toxicity Substance Identifier; ^2^ Chemical Abstract Services Registry Number; ^3^ Molecular Weight.

**Table 2:** Hematology parameters and methods.

Parameter	Method
Complete Blood Count	
White Blood Cells (WBCs)	Flow cytometry by two angles of light scatter Low angle: number of nuclei present High angle: nuclear complexity
Red Blood Cells (RBCs)	Flow cytometry by two angles of light scatter Low angle: size High angle: hemoglobin concentration
Hemoglobin (HGB)	Measured using cyanmethemoglobin
Hematocrit (HCT)	Calculated: (RBC × MCV) ÷ 10
Mean Corpuscular Volume (MCV)	Mean of RBC volume histogram
Mean Corpuscular Hemoglobin (MCH)	Calculated: (HGB ÷ RBC) × 10
Mean Corpuscular Hemoglobin Concentration (MCHC)	Calculated: (HGB ÷ [RBC × MCV]) × 1000
Mean Platelet Volume (MPV)	Flow cytometry by low-angle light scatter
Platelets	Flow cytometry by two angles of light scatter Low angle: size High angle: refractive index
Red Cell Distribution Width (RCDW)	Calculated: 100 × (Standard Deviation of RBC volume histogram ÷ MCV)
Leukocyte Differential Count	
Absolute Neutrophils (ABNEUTs)	Individual cell types initially determined as WBC percentages. Absolute cell counts were calculated by multiplying the total white blood cell count by the decimal expression of the percentage of the individual cell population.
Absolute Lymphocytes (ABSLYMs)	
Absolute Monocytes (ABSMONs)	
Absolute Eosinophils (ABEOSs)	
Absolute Basophils (ABSBASs)	
Reticulocyte Count	
Absolute Reticulocytes (ABRETis)	Calculated by multiplying the total red blood cell count by the decimal expression of the reticulocyte percentage.
Reticulocyte Percentage	Same as RBC plus zero-angle light scatter–light absorption (cells stained with oxazine 750 absorb more light than mature RBCs.)

**Table 3:** Clinical chemistry parameters and methods.

Parameter	Method
Albumin (ALB)	Bromocresol green (colorimetric)
Alkaline Phosphatase (ALPi)	p-Nitrophenyl-phosphate (bichromatic rate)
Alanine Aminotransferase (ALTi)	L-Alanine to Alpha Ketoglutarate (bichromatic rate)
Aspartate Aminotransferase (AST)	L-Aspartate to Alpha Ketoglutarate (bichromatic rate)
Blood Urea Nitrogen (BUN)	Urease (bichromatic rate)
Calcium (CA)	o-Cresolphthalein Complexone (colorimetric)
Cholesterol (CHOL)	Cholesterol Esters (colorimetric)
Creatine Kinase (CK)	Creatine Phosphate to Adenosine diphosphate (multiple point rate)
Chloride (CL)	Ion-selective Electrode (potentiometric)
Creatinine (CRE2)	Creatinine + Picrate (bichromatic rate)
Gamma Glutamyltransferase (GGT)	Gamma-glutamyl-3-carboxy-4-nitroanilide to Glycylglycine (bichromatic rate)
Glucose (GLUC)	Hexokinase–Glucose-6-phosphate Dehydrogenase (colorimetric)
Potassium (K)	Ion-selective Electrode (potentiometric)
Sodium (NA)	
Phosphorus (PHOS)	Phosphomolybdate (colorimetric)
Total Bilirubin (TBILI)	Diazo (colorimetric)
Total Protein (TPROT)	Biuret (colorimetric)
Triglycerides (TRIGs)	Glycerol-3-phosphate (colorimetric)

**Table 4:** Summary of observations from single-dose tolerability studies in male SD rats (*n* = 3/dose).

PFAS	Dosing Volume (mL/kg)	Dose Levels	Summary
PFNAC	10	0, 25, 75, 225, and 667 mg/kg in corn oil	No mortality and no abnormal clinical observations, some lowering of bodyweight, bodyweight changes, and feed consumption in all the treated groups not reaching statistical significance
PFHI	5	0, 30, 100, 300, and 1000 mg/kg in corn oil	No mortality and no abnormal clinical observations, no effect on bodyweight; some bodyweight changes in high-dose group were considered as chemical-related
MHFPK	10	0, 30, 100, 300, and 1000 mg/kg in corn oil	No mortality. Clinical signs of low activity and minimal ataxia on study day 1 at 1000 mg/kg, with recovery on the following two observation days
CTFPA	5	0, 30, 100, 300, and 1000 mg/kg in corn oil	No mortality and no effect on absolute bodyweight gains or water consumption; clinical observations of low activity, decreased bodyweight and decreased feed consumption at high doses

**Table 5:** Summary of the key substance-related findings in 14-day oral studies in male and female SD rats ^1,2^.

Chemical	PFNAC	PFHI	MHFPK	CTFPA
Doses (corn oil vehicle, dose volume of 5 mL/kg)	0, 10, 30, or 100 mg/kg-day	0, 30, 100, or 300 mg/kg-day	0, 30, 100, or 300 mg/kg-day	0, 30, 100, or 300 mg/kg-day
Morbidity and mortality	None	None	None	None
Clinical observations	None	None	None	None
Bodyweight and bodyweight gain	No changes	No changes	No changes	No changes
Feed consump-tion	No effects	Females: dose-dependent increase, not correlated with bodyweight changes; Males: no effects	No effects	No effects
Ophthalmology	No ocular lesions	No ocular lesions	No ocular lesions	No ocular lesions
Hematology	No effects considered as chemical related	Decreased red cell mass at 300 mg/kg-day in females and all the dose levels in males; minimal increases in absolute reticulocyte concentrations and from minimal to mild increases in MPV in both sexes at all the dose levels	No effects considered as chemical related	Mild increases in MPV in males at all the doses, not seen in females
Clinical chemistry	From mild to moderate decreases in mean cholesterol levels in males at all the dose levels and in females at 30 and 100 mg/kg-day, with from mild to moderate increases in triglycerides in both sexes at 100 mg/kg-day and in females at 30 mg/kg-day	Mild increases in cholesterol levels in both sexes at 300 mg/kg-day and mild increases in glucose in males only at ≥100 mg/kg-day; from minimal to moderate increases in phosphorus in both sexes in all the dose groups	Minimal increases in BUN and creatinine at 300 mg/kg-day for males; increased creatinine in females at ≥100 mg/kg-day	Females: mild increases in albumin, A/G ratio and mild sporadic decreases in globulin and minimal increases in the total protein; males: minimal increases in calcium and from minimal to mild increases in BUN at all the dose levels. At 300 mg/kg-day, mild increases in ALPi
Coagulation endpoints	No effects	No effects	No effects	No effects
Organ weight changes	Dose-proportional increases in absolute and relative liver weights in all exposed males and females	Increases in absolute and relative liver weights in males at 300 mg/kg-day and increases in liver weight/bodyweight ratio at ≥100 mg/kg-day; no changes in females	No test-substance-related changes	Females: liver at >100 mg/kg-day; males: liver, dose-dependent pattern in all the dose groups
Gross lesions	None	None	None	None
Histopathology	From minimal to moderate liver hypertrophy in males only in 2/5, 3/5, and 4/4 at doses of 10, 30, and 100 mg/kg-day, respectively	Mild liver hypertrophy in 4/5 males at 300 mg/kg-day and in 1/5 at 100 mg/kg-day; none in females	No test-substance-related changes	From minimal to moderate diffuse hepatocellular hypertrophy seen in the livers of males at ≥30 mg/kg-day (5/5 in all the groups, with severity increasing with dose) and females at ≥100 mg/kg-day (5/5 with, severity increasing with dose)

^1^ Effects were not considered as substance-related if they occurred sporadically within groups with no observed dose–response relationship, at comparable levels in control and dosed rats, or if a common incidental finding in this strain of rat (*n* = 5/sex/dose). ^2^ Abbreviations: MPV—mean platelet volume; BUN—blood urea nitrogen; A/G—albumin/globulin ratio; ALPi—alkaline phosphatase. Liver hypertrophy was from minimal to mild in the affected animals. Minimal hypertrophy is described as “tissue alterations that slightly exceed that which is considered within normal limits”. Mild hypertrophy is described as “tissue alterations are easily identified but of limited or minor severity”. Moderate hypertrophy is described as “tissue alterations are prominent but still have considerable potential for increased severity”.

**Table 6:** Descriptive results for subchronic 90-day oral PFAS studies ^1^.

Chemical	PFNAC	PFHI	MHFPK	CTFPA
Doses (corn oil vehicle, dose rate of 3 mL/kg)	0, 0.1, 0.3, 1.0, 3.0, and 10 mg/kg-day for male rats; 0, 0.3, 1.0, 3.0, 10, and 30 mg/kg-day for female rats	0, 12.5, 25, 50, 100, and 200 mg/kg-day	0, 18.8, 37.5, 75, 150, and 300 mg/kg-day	0, 1.9, 3.8, 7.5, 15, and 30 mg/kg-day for male rats; 0, 6.3, 12.5, 25, 50, and 100 mg/kg-day for female rats
Morbidity and mortality	None	None	None	None
Clinical observations	None	None	None	None
Bodyweight (BW) and bodyweight gain	None in females; effects in males at 10 mg/kg-day were not considered adverse because they did not persist through the study (days 8–29)	None in females; dose-dependent reduced BW changes in males in all the dose groups, but BW gain on SDs 1-90 was not significantly affected	No changes in BW or BW gain over the course of the study	None CTPFA related
Feed consumption	Increased in males and females in some dose groups during some time periods, not considered as adverse (not persistent or consistent although sometimes statistically significant)	No changes in males; increased feed consumption in females at 25, 50, and 100 mg/kg-day but not at 200 mg/kg-day	No changes in males; some statistically significant changes in females, not considered as compound related	Significant non-adverse increases in males at ≥3.8 mg/kg-day; minor effects in females, not considered as CTFPA related
Hematology	No changes in females; Males—minimal decreases in red cell mass (HGB, HCT, and RBC) at ≥0.1 mg/kg-day, minimal decreases in absolute reticulocytes at ≥1 mg/kg and from minimal to mild decreases in neutrophils and eosinophils at ≥0.1 mg/kg-day ^2^	Minimal decreases in reticulocytes in males at ≥50 mg/kg-day; minimal increases in MPV in females and sporadically in males at 200 mg/kg-day and in both sexes at 50 and 100 mg/kg-day	From minimal to mild dose-dependent increases in MPV in both sexes at ≥18.8 mg/kg-day	From minimal to mild decreases in red cell mass (RBC, HCT, and HGB) and increases in RDW in males at ≥7.5 mg/kg-day; no similar effect in females; from minimal to mild increased MPV in males at ≥3.8 mg/kg-day and in females at ≥50 mg/kg-day
Ophthalmology	No ocular lesions	No ocular lesions	No ocular lesions	No ocular lesions
Functional observational battery	Some changes, all considered as incidental and non-adverse	No changes	No changes	No changes
Locomotor activity	No effects	No effects	No effects	No effects
Clinical chemistry	Mild decreases in cholesterol and mild increases in glucose in males at ≥0.1 mg/kg-day and in females at 30 mg/kg ^3^; from mild to moderate increases in ALPi in males at only ≥ 0.3 mg/kg-day and from minimal to mild globulin decreases in males at only at ≥1.0 mg/kg-day; mild increases in BUN and albumin in males only at ≥1.0 mg/kg-day, resulting in increased albumin/globulin (A/G) ratios ^4^	From minimal to mild dose-dependent increases in cholesterol in males at ≥25 mg/kg-day and in females at 200 mg/kg-day; mild increases in the total protein, resulting from increases in both albumin and globulin in females at 200 mg/kg-day; males at all the dose levels had mild decreases in calcium, phosphorus, potassium, and/or CK—not a dose-dependent relationship and likely indirect effects related to decreases in bodyweight gains	From sporadic mild to moderate increases in triglycerides in males at ≥100 mg/kg-day, and the relationship to compound administration is unclear; in females, only at 18.8 mg/kg-day, sporadic variations in albumin, globulin, the total protein, and the A/G ratio, with the relationship to MHFPK administration judged as unclear	From minimal to mild increases in albumin and the A/G ratio in males at ≥7.5 mg/kg-day and in females at ≥50 mg/kg; only in males—from minimal to mild decreases in globulin at ≥7.5 mg/kg-day; from minimal to mild increases in ALPi at ≥7.5 mg/kg-day, and minimally increased BUN at 30 mg/kg-day
Coagulation endpoints	Males at ≥1 mg/kg-day, mildly shortened APTT, not considered as biologically significant	Dose-dependent minimal prolongations in PT (in males at ≥12.5 mg/kg-day and in females at ≥25 mg/kg-day	Males at ≥37.5 mg/kg-day, from minimal to mild dose-dependent prolongations in PT; no effects in females	From minimal to mild dose-dependent decreased APTT in males at ≥3.8 mg/kg; no effect in females
Organ weight changes	Mean absolute and/or relative organ weight increases in the liver and kidneys of males at ≥0.3 mg/kg-day and females at ≥3 mg/kg-day	Mean absolute and/or relative dose-dependent organ weight increases in the liver of females at ≥100 mg/kg-day and males at ≥25 mg/kg-day and kidney weight increases only in males at ≥100 mg/kg-day ^5^	None considered as MHFPK related	Mean absolute and/or relative liver weight increases in males at ≥3.8 mg/kg-day and in females at ≥25 mg/kg-day ^6^; mean thymus weights increased in males at ≥15 mg/kg-day; mean kidney weights increased in females at ≥50 mg/kg-day
Gross lesions	None considered as PFNAC related	None considered as PFHI related	None considered as MHFPK related	None considered as CTFPA related
Histopathology ^7^	The kidney and liver ^4^ of males at ≥0.3 mg/kg-day and females at 30 mg/kg-day; the thyroid gland in males	The kidney and liver ^5^ of males at ≥100 mg/kg-day and the liver of females at 200 mg/kg-day	None considered as MHFPK related	The liver of males and females; the heart and nasal turbinates of males

^1^ Only effects considered as compound related. Effects may not be considered as compound related if they were sporadic, not dose-dependent, very small, or consistent with normal biologic or analytic variation. ^2^ Overall findings suggestive of decreased hematopoiesis within bone marrow but lacked microscopic correlates. ^3^ Changes in glucose and cholesterol suggestive of altered energy metabolism. ^4^ From minimal to mild decreases in AST and/or CK in most male and female dose groups not considered as directly PFNAC related. Findings among glucose, ALPi, BUN, albumin, and globulin correlated with, and may have been related to, increased organ weights and microscopic findings in the kidneys and liver; kidney—minimal tubule epithelial hypertrophy in a dose-dependent pattern of increasing incidence in males at ≥0.3 mg/kg-day and in females at 30 mg/kg-day only; liver—from minimal to moderate centrilobular or panlobular hepatocellular hypertrophy in a generally dose-dependent pattern of increasing incidence and severity in males at ≥0.3 mg/kg-day and minimal severity in females at 30 mg/kg-day only, with from minimal to mild sporadic focal hepatocellular necrosis in a non-dose-dependent pattern. ^5^ Liver weight increases corresponded to centrilobular liver hypertrophy in males at 100 and 200 mg/kg-day and in females at 200 mg/kg-day. Kidney weight increases corresponded to findings of increased incidence and severity of chronic progressive nephropathy and hyaline droplet accumulation in affected groups. Additional histopathology findings included minimal mononuclear cell infiltrates in the cecum of males at ≥25 mg/kg-day and in females at ≥100 mg/kg-day and mixed infiltrates surrounding the ducts of the submandibular salivary gland in males at ≥50 mg/kg-day and in females at ≥100 mg/kg-day. ^6^ This correlated with microscopic findings of from minimal to mild hepatocellular hypertrophy occurring in a generally dose-dependent pattern of increasing severity and incidence in males at ≥3.8 mg/kg-day and in females at ≥50 mg/kg-day. There was increased incidence and severity of from minimal to mild rodent progressive cardiomyopathy in males at 15 and 30 mg/kg-day, and males at 30 mg/kg-day had increased incidence of minimal mixed cell inflammation of the nasal turbinates and nasal passages. ^7^ Minimal hypertrophy is described as “tissue alterations that slightly exceed that which is considered within normal limits”. Mild hypertrophy is described as “tissue alterations are easily identified but of limited or minor severity”. Moderate hypertrophy is described as “tissue alterations are prominent but still have considerable potential for increased severity”.

**Table 7:** Comparative PFAS response summary in the liver and kidney following BMD response analysis.

Response	PFAS	Male Rat (Dose in mg/kg-day)				Female Rat (Dose in mg/kg-day)			
		NOEL	LOEL	BMD10	BMDL	NOEL	LOEL	BMD10	BMDL
Relative liver weight increase	PFNAC	0.1	0.3	0.15	0.13	10	30	12.8	10.1
	PFHI	12.5	25	ND	ND	50	100	ND	ND
	CTFPA	1.9	3.8	ND	ND	12.5	25	ND	ND
	MHFPK	NR	NR	ND	ND	NR	NR	ND	ND
Absolute liver weight increase	PFNAC	0.1	0.3	0.09	0.04	10	30	11.2	8.8
	PFHI	50	100	ND	ND	100	200	57.8	8.1
	CTFPA	3.8	7.5	52.7	11.6	12.5	25	13.6	5.1
	MHFPK	NR	NR	ND	ND	NR	NR	ND	ND
Relative kidney weight increase	PFNAC	0.3	1.0	ND	ND	1	3	6.0	0.8 *
	PFHI	50	100	69.7	56.6	NR	NR	ND	ND
	CTFPA	NR	NR	ND	ND	NR	NR	ND	ND
	MHFPK	NR	NR	ND	ND	NR	NR	ND	ND
Absolute kidney weight increase	PFNAC	0.1	0.3	0.17	0.06	3	10	13.4	2.68
	PFHI	100	200	122	83.8	NR	NR	ND	ND
	CTFPA	NR	NR	ND	ND	NR	NR	ND	ND
	MHFPK	NR	NR	ND	ND	NR	NR	ND	ND

NR—no response, no statistically significant dose-dependent changes; ND—not determined, data unsuitable for BMD analysis or no best fit model identified; * Uncertain validity due to the difference between BMD10 and BMDL (95%) being greater than fivefold.

**Table 8:** The 14-day study’ bioanalytical results—parent chemical measurements in rat plasma (mean ± standard deviation, N = 10) ^1^.

PFAS	Dose mg/kg-day	Male Rat on Day 1 ^2^ (ng/mL)	Male Rat on Day 15 (ng/mL)	Female Rat on Day 1 (ng/mL)	Female Rat on Day 15 (ng/mL)
PFNAC ^3^	0, 10, 30, and 100	Parent chemical not detected at the highest dose	Parent chemical not detected at the highest dose	Parent chemical not detected at the highest dose	Parent chemical not detected at the highest dose
PFHI ^4^	0	ND/BLOD	ND	ND	ND
	30	159 ± 79.3	<BLOD/≤LOQ	14.7 ± 8.21	ND/BLOD
	100	333 ± 147	4.87 ± 1.93	470 ± 427	≤LOQ
	300	2040 ± 1250	26.7 ± 8.79	1070 ± 689	14.3 ± 7.12
CTFPA ^5^	0	ND	43.5 ± 32.3	ND	10.3 ± 4.65
	30	48,400 ± 8430	49,800 ± 15,800	46,700 ± 10,000	27,200 ± 6150
	100	122,000 ± 43,800	99,100 ± 53,500	107,000 ± 30,400	45,400 ± 17,400
	300	321,000 ± 60,600	120,000 ± 41,100	297,000 ± 56,500	123,000 ± 39,700

^1^ MHFPK was not measured due to the lack of availability of a method suitable for plasma. ^2^ Sample was collected 2 hours post dosing. ^3^ Parent chemical was not detected in any representative samples analyzed (at the highest dose level) at 1 or 15 days, indicating very rapid metabolism. Single large peak identified that is likely associated with a metabolite; LOD = 0.7 ng/mL. ^4^ ND—not detected, BLOD—below the limit of detection of 1.7 ng/mL, LOQ—the limit of quantification (5 ng/mL). ^5^ LOD is 1.72 ng/mL.

**Table 9:** Subchronic study’s bioanalytical results—parent chemical measurements in rat plasma (mean ± standard deviation, N = 10) ^1^.

PFAS	Male Rat on Day 91		Female Rat on Day 91	
	Dose mg/kg-day	Concentration (ng/mL)	Dose mg/kg-day	Concentration (ng/mL)
PFNAC ^2^	0, 0.1, 0.3, 1.0, 3.0, and 10	Parent chemical not detected at the highest dose	0, 0.3, 1.0, 3.0, 10, and 30	Parent chemical not detected at the highest dose
PFHI ^3^	0	ND	0	ND
	12.5	BLOD	12.5	BLOD
	25	<BLOD/≤LOQ	25	<BLOD/≤LOQ
	50	14.0 ± 6.21	50	12.2 ± 9.91
	100	43.8 ± 7.53	100	48.3 ± 22.9
	200	52.4 ± 24.1	200	76.7 ± 24.1
CTFPA ^4^	0	<BLOD/≤LOQ	0	<BLOD/≤LOQ
	1.9	12,616 ± 5004	6.3	12,646 ± 3192
	3.8	22,318 ± 12,129	12.5	24,720 ± 8538
	7.5	43,320 ± 17,245	25	50,960 ± 16,608
	15	62,111 ± 29,468	50	71,480 ± 35,696
	30	84,350 ± 21,516	100	116,240 ± 42,239

^1^ MHFPK was not measured due to the lack of availability of a method suitable for plasma. ^2^ Parent chemical was not detected in any representative samples analyzed (at the highest dose level). Single large peak identified that is likely associated with a metabolite or analog; LOD = 0.7 ng/mL. ^3^ ND—Not detected, BLOD—below the limit of detection—1.7 ng/mL, LOQ—limit of quantitation—5 ng/mL. ^4^ BLOD—5 ng/mL, LOQ—10 ng/mL; trace levels measured in the plasma of the control rats likely reflect environmental contamination.

## Data Availability

The study reports are available at https://doi.org/10.23645/epacomptox.29171192 (accessed on 3 June 2025).

## Supplementary Materials

The following supporting information can be downloaded at https://www.mdpi.com/article/10.3390/toxics13070524/s1: Table S1. Gas chromatography (GC) analytical conditions for PFHI-, PFNAC-, and MHFPK-dosing formulations; Table S2. High-performance liquid chromatography (HPLC) analytical conditions for CTFPA-dosing formulation; Table S3. GC–mass spectrometry (GC-MS) analytical conditions for PFNAC and PFHI; Table S4. Ultra-performance liquid chromatography (UPLC)-MS analytical conditions for CTFPA; Table S5. Summary of bodyweight (BW) gain and feed consumption data in male SD rats (from day 1 to day 90); Table S6. Summary of bodyweight (BW) gain and feed consumption data in female SD rats (from day 1 to day 90); Table S7. Summary of liver weight data in male and female SD rats in subchronic studies; Table S8. Summary of kidney weight data in male and female SD rats in subchronic studies; Table S9. Summary of coagulation data; Table S10. Summary of selected hematology data in male SD rats; Table S11. Summary of selected hematology data in female SD rats; Table S12. Summary of selected clinical chemistry data in male SD rats; Table S13. Summary of selected clinical chemistry data in female SD rats.

## Abbreviations

The following abbreviations are used in this manuscript:

PFASs	Per- and polyfluoroalkyl substances
PFOA	Perfluorooctanoic acid
PFOS	Perfluorosulfonic acid
PFNAC	1H,1H,9H-perfluorononyl acrylate
PFHI	1H,1H,2H,2H-perfluorohexyl iodide
CTFPA	2-chloro-2,3,3,3-tetrafluoropropionic acid
MHFPK	3,3,4,4,5,5,5-heptafluoropentan-2-one
NAMs	New approach methodologies
GC	Gas chromatography
FID	Flame ionization detector
HPLC	High-performance liquid chromatography
MS	Mass spectrometry
DI	Deionized
PHS	Public Health Service
AAALAC	Association for the Assessment and Accreditation of Laboratory Animal Care
BW	Bodyweight
BWCs	Bodyweight changes
FC	Feed consumption
WC	Water consumption
FOB	Functional observational battery
LMA	Locomotor activity
PTT	Prothrombin time
APPT	Activated partial prothrombin time
NBF	Neutral buffered formalin
EDTA	Ethylenediaminetetraacetic acid
LOEL	Lowest observed effect level
NOEL	No observed effect level
BMD	Benchmark dose
HGB	Hemoglobin
HCT	Hematocrit
RBCs	Red blood cells
ABRETis	Absolute numbers of reticulocytes
MPV	Mean platelet volume
ALPi	Alkaline phosphatase
ALB	Albumin
GLOB	Globulin
A/G	Albumin/globulin ratio
CK	Creatinine kinase
NTP	National toxicology program
NHANES	National Health and Nutrition Examination Survey
U.S. EPA	U.S. Environmental Protection Agency
