# Supplementary material for: Subchronic Toxicities of Four Per- and Polyfluoroalkyl Substances (PFASs) by Oral Exposure in Sprague–Dawley Rats
Source: Toxics. 2025 Jun 22;13(7):524. doi: 10.3390/toxics13070524 (PMC12298827; doi:10.3390/toxics13070524)
Supplement: Supplementary file 1 [file toxics-13-00524-s001.zip › toxics-3648202-supplementary.pdf]

Table S1. Gas chromatography (GC) analytical conditions for PFHI, PFNAC and MHFPK dosing formulations

Chemical	PFNAC	PFHI	MHFPK
System	Agilent 6890N (Agilent Technologies, Santa Clara, CA)		
Data System	Empower 3, Build 3471, (Waters Corp., Milford, MA)		
Column	Agilent J&W DB-624 (30 m x 0.32 mm ID, 1.8 μm film)		
Carrier Gas Flow Rate	Helium 2 mL/min		
Oven Temperature	Initial temp at 40 °C for 2 min, ramp to 250 °C at 20 °C/min, hold at 250 °C for 2.5 min (15 min total)		Initial temp at 30 °C for 2 min, ramp to 250 ◦C at 20 °C/min, hold at 250 °C for 2.5 min (16.5 min total)
Injection Type (ratio)	Split (~20:1)		
Injection volume	2 μL	2 μL	1 μL
Injector Temperature	200 °C		
Injection Solvent	dichloromethane		
Retention Time (RT) Chemical	9.6 min	6.9 min	3.5 min
Internal Standard RT	6.9 min	9.6 min	Not applicable
Detector Temperature	FID / 300 °C		

Table S2. High performance liquid chromatography (HPLC) analytical condition for CTFPA dosing formulation

Instrument	LC-302 Waters ARC System with PDA (Waters Corp, Milford, MA)		
Data System	Empower 3, Build 3471, Feature Release 6 Service Pack, Version 1(Waters Corp., Milford, MA)		
Analytical Column	Waters XBridge C18, 3.5 μm, 4.6 x 150 mm		
Column Temperature	40 ° C		
Sample Temperature	15° C		
Injection Volume	50 μL		
Mobile Phases	A: 10 mM ammonium acetate in methanol: water (5:95, v:v)		
	B: 10 mM ammonium acetate in methanol: water (95:5, v:v)		
Flow Rate	1 mL/min		
Solvent Gradient	Time (minutes)	% A	% B
	0.0	80	20
	2.0	80	20
	4.0	0	100
	5.0	0	100
	6.0	80	20
	10	80	20
PDA Settings	205 nm extracted		
Retention Time	~ 3.2 min		

Table S3. GC-mass spectrometry (GC-MS) analytical conditions for PFNAC and PFHI

PFAS Chemical	PFNAC	PFHI
GC-MS System	7890 GC/ 5975 MSD (Agilent Technologies, Santa Clara, CA)	
Data Software	MSD Masshunter Version: 10.0.380 (Agilent Technologies, Santa Clara, CA)	
Headspace Autosampler	Combipal Autosampler (CTC Analytics, Zwingen, Switzerland)	
Vial Size	2 mL	
Syringe Volume / Sample Volume	10 μL / 1μL	
Column	Agilent J&W DB-624 (30 m x 0.32 mm ID, 1.8 μm film)	
Carrier Gas	Helium 2 mL/min	
Oven Temperature Program	Initial temp at 40 °C for 2 min, ramp to 240 °C at 20 °C/min, hold at 240 °C for 3 min (15 min total)	
Injector Temperature	200 °C	
Injection Mode	splitless	
Retention Time	~8.51 min	~5.87 min
Auxiliary Temperature	250 °C	
MS Source Temperature	250 °C	
Quadruple Temperature	150 °C	
MS Ionization Model	Electron Ionization (70 eV)	
Single Ion Monitoring Acquisition Mode	m/z 131<br>m/z 181 for confirmation only	m/z 374<br>m/z 141 for confirmation only

Table S4. Ultra-performance liquid chromatography (UPLC)-MS analytical conditions for CTFPA

<table>
<tr><td>Instrument</td><td colspan="3">Waters Acquity UPLC modified for low PFAS background (Waters Corp., Milford, MA) SCIEX API 5000</td></tr>
<tr><td>Analytical Column</td><td colspan="3">Waters Acquity, BEH C18, 1.7 μm, 2.1 x 100 mm</td></tr>
<tr><td>Guard Column</td><td colspan="3">Waters Vanguard, BEH C18, 1.7 μm, 2.1 x 5 mm</td></tr>
<tr><td>Isolator Column</td><td colspan="3">Waters 2.1 x 50 mm</td></tr>
<tr><td>Column Temperature</td><td colspan="3">40 ˚C</td></tr>
<tr><td>Sample Temperature</td><td colspan="3">15 ˚C</td></tr>
<tr><td>Injection Volume</td><td colspan="3">2 μL</td></tr>
<tr><td>Autosampler Wash</td><td colspan="3">Seal/Weak: water/methanol (90:10, v:v); strong needle wash: methanol</td></tr>
<tr><td>Mobile Phases</td><td colspan="3">A: 10 mM ammonium acetate in water:methanol (95:5, v:v)
B: 10 mM ammonium acetate in methanol: water (95:5, v:v)</td></tr>
<tr><td>Flow Rate</td><td colspan="3">2 mL/min</td></tr>
<tr><td rowspan="7">Solvent Gradient</td><td>Time (min)</td><td>%A</td><td>%B</td></tr>
<tr><td>0.0</td><td>90</td><td>10</td></tr>
<tr><td>1.0</td><td>90</td><td>10</td></tr>
<tr><td>8.5</td><td>0</td><td>100</td></tr>
<tr><td>9.5</td><td>0</td><td>100</td></tr>
<tr><td>9.6</td><td>90</td><td>10</td></tr>
<tr><td>12.5</td><td>90</td><td>10</td></tr>
<tr><td rowspan="6">MS Source Parameters</td><td>Gas 1, Gas2</td><td colspan="2">40 psi, 70 psi</td></tr>
<tr><td>Curtain Gas</td><td colspan="2">40 psi</td></tr>
<tr><td>CAD Gas</td><td colspan="2">10 psi</td></tr>
<tr><td>Source Temperature</td><td colspan="2">300 ˚C</td></tr>
<tr><td>Spray Voltage (Neg ESI)</td><td colspan="2">-2500 V</td></tr>
<tr><td>EP, CXP</td><td colspan="2">-10 V, -12V</td></tr>
<tr><td rowspan="3">Detection</td><td>Compound</td><td>Mass Transitions Collision Energy (V)*</td><td>Retention Time (min)</td></tr>
<tr><td>CFTPA</td><td>Quant - 178.9 > 134.9 (-12)
Qual – 180.9 > 136.9 (-13)</td><td>~ 2.46</td></tr>
<tr><td>Internal Standard**</td><td>433.0 > 412.0 (-34)</td><td>~8.0</td></tr>
</table>

*Collision merges indicated in (). **1H,1H, 2H, 2H perfluorooctane sulfonate sodium salt ($^{13}C_2$, D_4), FTS-$^{13}C_2D$

Table S5. Summary of body weight (BW) gain and feed consumption data in male SD rats (day 1 to day 90)[1]

PFAS	Dose(mg/kg-day)	Total BW change(g)	% Diff	% BW Gain (%)	% Diff	Total Food Consumed (g)	% Diff
PFNAC	0	301.81±47.09	-	100.97±11.84	-	2219±223	-
	0.1	333.40±70.59	10.47	110.97±15.40	9.90	2298±225	3.57
	0.3	339.42±52.88	12.46	113.17±14.14	12.08	2427±208	9.40
	1.0	314.97±62.87	4.36	108.93±26.57	7.88	2357±89.7	6.25
	3.0	266.69±58.12	-11.64	89.59±21.30	-11.27	2381±114	7.33
	10	238.64±76.86	-20.93	80.97±24.88	-19.80	2270±227	2.34
PFHI	0	325.62±44.04	-	113.16±13.33	-	2376±197	-
	12.5	319.83±38.29	-1.78	114.45±25.75	0.87	2217±70.6	-6.69
	25	321.29±36.08	-1.33	112.88±12.91	-0.52	2266±233	-4.60
	50	307.16±32.98	-5.67	108.23±12.57	-4.61	2229±160	-6.15
	100	294.89±34.07	-9.44	106.43±17.95	-6.20	2241±168	-5.67
	200	279.08±47.04*[1]	-14.29	100.73±18.59	-11.23	2179±146	-8.27
CTFPA	0	316.71±39.28	-	125.39±16.27	-	2067±130	-
	1.9	316.63±36.90	-0.03	125.06±20.68	-0.26	2084±131	0.81
	3.8	354.57±60.89	11.95	137.76±22.45	9.87	2255±177	9.08*
	7.5	376.00±68.85	18.72	145.16±20.48	15.76	2345±128	13.5**
	15	342.33±37.60	8.09	135.14±18.23	7.78	2368±125	14.6**
	30	314.42±74.00	-0.72	121.40±22.69	-3.18	2281±167	10.33**
MHFPK	0	293.7±2.29	-	97.84±13.77	-	2176±38.1	-
	18.8	300.42±45.96	2.29	103.77±24.34	6.06	2143±130	-1.53
	37.5	302.87±20.80	3.12	101.81±12.57	4.06	2133±76.7	-2.01
	75	308.68±62.17	5.10	102.83±26.83	5.10	2218±163	1.92
	150	313.14±57.41	6.62	104.78±27.74	7.09	2253±108	3.52
	300	300.61±76.65	2.35	102.36±32.89	4.62	2196±177	0.89

[1]Data presented as mean ± SD. % Diff is % difference from control. N =10/group unless otherwise indicated.

* Anova & Dunnett * = $p < 0.05$, ** = $p < 0.01$

Table S6. Summary of body weight (BW) gain and feed consumption data in female SD rats (day 1 to day 90)[1]

PFAS	Dose(mg/kg-day)	Total BW change(g)	% Diff	% BW Gain (%)	% Diff	Total Food Consumed (g)	% Diff
PFNAC	0	101.14±18.72	-	48.79±8.45	-	1407±150	-
	0.3	98.25±15.26	-2.86	47.16±6.48	-3.35	1466±127	4.16
	1.0	94.80±17.12	-6.27	45.99±8.25	-5.73	1444±69.71	2.64
	3.0	99.10±9.40	-2.02	47.09±4.71	-3.49	1512±49.1	7.48
	10	95.48±15.20	-5.60	46.00±7.26	-5.72	1479±20.0	5.11
	30	106.79±18.99	5.59	52.06±10.70	6.69	1525±125*	8.40
PFHI	0	123.29±30.75	-	60.54±12.52	-	1472±82.3	-
	12.5	124.60±16.35	1.06	61.51±7.78	1.61	1474±70.4	0.12
	25	125.38±25.42	1.70	60.67±11.62	0.22	1579±66.7*	7.27
	50	132.23±21.27	7.25	65.42±13.29	8.07	1595±32.6*	8.31
	100	129.49±31.71	5.03	62.41±11.82	3.09	1671±184**	13.5
	200	108.57±17.71	-11.94	53.53±8.27	-11.58	1566±94.9	6.33
CTFPA	0	114.07±19.58	-	52.68±11.60	-	1543±39.6	-
	6.3	129.35±21.56	13.40	59.29±8.61	12.55	1671±36.8**	8.28
	12.5	119.91±32.16	5.12	56.17±16.95	6.64	1559±122	1.02
	25	115.80±18.89	1.52	53.36±10.16	1.29	1589±31.0	2.96
	50	113.68±18.80	-0.34	51.94±7.89	-1.40	1577±76.4	2.22
	100	107.34±10.68	-5.90	49.85±7.13	-5.37	1615±37.4**	4.68
MHFPK	0	91.05±20.74	-	37.76±8.23	-	1458±94.8	-
	18.8	115.68±31.68	27.05	47.40±10.19	25.52	1629±174*[1]	11.67
	37.5	107.92±35.09	18.53	44.49±11.99	17.83	1480±107	1.46
	75	94.83±12.51	4.15	39.38±5.47	4.28	1522±107	4.34
	150	90.34±21.13	-0.78	37.70±10.33	-0.15	1542±141	5.76
	300	95.70±17.30	5.11	39.88±6.85	5.61	1575±83.0	7.99

[1]Data presented as mean ± SD

*Kruskal-Wallis & Dunnett on ranks *= p < 0.05, **= p < 0.01 *[1] Anova & Dunnett * = p < 0.05, ** = p < 0.01

Table S7. Summary of liver weight data in male and female SD Rats in subchronic studies

PFAS	Male Rats				Female Rats			
	Dose (mg/kg-day)	Liver Weight (g)	Liver/Body Weight (%)	Liver/Brain Weight (%)	Dose (mg/kg-day)	Liver Weight (g)	Liver/Body Weight (%)	Liver/Brain Weight (%)
PFNAC	0	13.49 ±1.84	2.31 ± 0.10	602 ±96.5	0	7.71±0.88	2.59±0.25	389±53.6
	0.1	14.99 ±2.67	2.41 ±0.10	673 ±131	0.3	7.76±0.85	2.62±0.21	408±62.4
	0.3	17.52 ±1.87	2.82 ±0.18	793 ±102	1.0	7.89±1.20	2.67±0.27	412±55.7
	1.0	20.59±2.59	3.48±0.22	949±143	3.0	7.96±0.67	2.67±0.25	396±33.1
	3.0	20.96±2.98	3.81±0.30	966±148	10	8.42±0.59	2.88±0.26	419±33.6
	10	21.12±2.04	4.16±0.55	1016±117	30	9.83±1.09	3.22±0.33	499±60.4
PFHI	0	15.43±3.79	2.54±0.37	691±163	0	8.11±1.17	2.58±0.12	383±46.6
	12.5	15.35±1.07	2.59±0.17	706±55.8	12.5	8.53±1.79	2.68±0.42	416±90.9
	25	16.98±3.62	2.85±0.49	780±143	25	8.66±0.80	2.71±0.14	417±40.0
	50	17.05±1.53	2.92±0.19	754±92.4	50	8.92±0.80	2.76±0.18	454±57.4
	100	18.32±2.02	3.24±0.17	843±86.0	100	9.17±1.20	2.85±0.19	447±59.7
	200	20.17±2.91	3.70±0.38	944±136	200	9.65±1.37	3.20±0.25	481±75.0
CTFPA	0	13.36±2.07	2.40±0.22	599±89.4	0	8.36±0.83	2.56±0.16	417±43.8
	1.9	14.47±1.59	2.58±0.19	697±99.6	6.3	8.76±1.24	2.59±0.34	437±50.6
	3.8	17.18±4.01	2.85±0.50	780±167	12.5	8.97±0.97	2.71±0.12	428±52.8
	7.5	20.01±3.40	3.24±0.36	912±138	25	9.97±1.42	3.02±0.30	483±78.9
	15	20.28±3.40	3.47±0.51	873±140	50	10.49±1.33	3.21±0.28	520±103
	30	21.52±4.87	3.82±0.48	954±194	100	11.31±1.50	3.57±0.41	575±84.6
MHFPK	0	14.29±1.60	2.45±0.19	641±66.9	0	8.89±1.58	2.73±0.39	445±93.0
	18.8	14.06±1.91	2.41±0.14	649±82.7	18.8	8.74±1.10	2.51±0.16	435±45.8
	37.5	13.83±1.20	2.34±0.14	627±60.4	37.5	8.76±1.63	2.58±0.25	433±70.0
	75	15.73±1.54	2.62±0.24	706±73.8	75	8.53±1.08	2.62±0.37	432±61.3
	150	15.50±2.18	2.54±0.21	709±120	150	8.84±0.75	2.74±0.29	462±67.9
	300	15.58±3.41	2.61±0.26	693±155	300	8.59±0.90	2.63±0.21	430±50.4

Significantly Different from Control $p < 0.05$

Table S8. Summary of kidney weight data in male and female SD rats in subchronic studies

PFAS	Male Rats				Female Rats			
	Dose (mg/kg-day)	Kidney Weight (g)	Kidney/Body Weight (%)	Kidney/Brain Weight (%)	Dose (mg/kg-day)	Kidney Weight (g)	Kidney/Body Weight (%)	Kidney/Brain Weight (%)
PFNAC	0	2.98±0.25	0.52±0.05	133±14.4	0	1.66±0.14	0.56±0.05	83.6±8.61
	0.1	3.14±0.47	0.51±0.03	141±23.4	0.3	1.74±0.18	0.59±0.05	91.4±11.8
	0.3	3.46±0.32	0.56±0.02	157±17.0	1.0	1.73±0.16	0.59±0.05	90.7±8.10
	1.0	3.57±0.23	0.61±0.04	164±14.9	3.0	1.83±0.12	0.61±0.05	91.1±5.24
	3.0	3.57±0.47	0.65±0.04	164±23.3	10	1.81±0.07	0.62±0.06	89.98±5.90
	10	3.53±0.39	0.69±0.08	170±23.0	30	1.99±0.17	0.65±0.05	101±9.43
PFHI	0	3.14±0.38	0.52±0.04	141±15.5	0	1.78±0.15	0.57±0.05	84.2±7.49
	12.5	3.04±0.26	0.51±0.06	140±13.0	12.5	1.83±0.16	0.58±0.03	89.5±10.1
	25	3.20±0.40	0.54±0.06	147±13.2	25	1.83±0.18	0.57±0.03	88.2±9.33
	50	3.25±0.35	0.56±0.04	143.1±11.9	50	1.85±0.15	0.57±0.03	93.9±9.83
	100	3.38±0.34	0.60±0.05	156±16.0	100	1.84±0.18	0.58±0.06	89.7±9.01
	200	3.60±0.39	0.66±0.05	168±16.8	200	1.83±0.18	0.61±0.04	91.3±10.0
CTFPA	0	3.01±0.53	0.54±0.07	135±18.6	0	1.83±0.17	0.56±0.04	91.0±6.47
	1.9	3.12±0.24	0.56±0.05	150±12.9	6.3	1.91±0.23	0.57±0.08	95.5±12.2
	3.8	3.35±0.52	0.56±0.06	152±22.5	12.5	1.97±0.20	0.60±0.04	93.7±9.46
	7.5	3.47±0.43	0.56±0.05	159±24.5	25	1.97±0.16	0.60±0.06	95.0±9.72
	15	3.40±0.23	0.58±0.04	147±11.2	50	2.07±0.15	0.63±0.04	102±13.7
	30	3.35±0.40	0.60±0.06	148±9.45	100	2.05±0.22	0.65±0.06	104±10.1
MHFPK	0	3.12±0.28	0.54±0.04	140±13.3	0	1.91±0.17	0.59±0.05	95.4±9.71
	18.8	3.11±0.38	0.54±0.05	144±18.8	18.8	1.94±0.18	0.56±0.04	96.5±7.20
	37.5	2.95±0.28	0.50±0.02	134±14.3	37.5	1.84±0.20	0.55±0.05	91.4±11.0
	75	3.25±0.26	0.54±0.04	146±9.68	75	1.91±0.10	0.59±0.06	96.8±6.31
	150	3.28±0.41	0.54±0.05	150±22.2	150	1.84±0.10	0.57±0.03	95.8±8.51
	300	3.24±0.32	0.55±0.04	144±13.5	300	1.94±0.20	0.59±0.05	97.1±10.9

Significantly Different from Control $p < 0.05$.

Table S9. Summary of coagulation data[1]

PFAS	Male Rats			Female Rats		
	Dose (mg/kg-day)	PT (sec)[2]	APTT (sec)[3]	Dose (mg/kg-day)	PT (sec)[2]	APTT (sec)[3]
PFNAC	0	14.61±0.46	20.58±7.45	0	13.51±0.50	21.30±9.10
	0.1	15.98±1.13**	23.16±8.72	0.3	13.79±0.47	21.25±7.30
	0.3	15.10±0.81	15.99±5.23	1.0	14.03±0.67	20.13±7.44
	1.0	15.56±0.92	12.70±1.47**	3.0	14.15±0.38*	20.34±8.08
	3.0	14.98±0.66	12.24±0.48**	10	14.11±0.43*	22.50±8.23
	10	15.81±1.16*	12.57±1.30**	30	14.44±8.23**	22.09±10.55
PFHI	0	14.58±0.27	22.63±6.87	0	13.33±0.35	18.63±8.66
	12.5	14.91±0.56	23.37±10.14	12.5	13.34±0.72	18.80±13.32
	25	14.69±0.92	29.06±9.55	25	13.51±0.49	18.59±7.98
	50	15.14±0.74	20.03±6.26	50	13.48±0.57	20.46±8.28
	100	15.16±0.28	26.73±5.36	100	13.79±0.58	17.57±7.77
	200	15.32±0.82	25.80±6.24	200	13.98±0.66	19.34±6.54
CTFPA	0	15.24±0.71	18.82±3.65	0	14.41±0.61	14.74±4.08
	1.9	15.55±0.61	18.83±5.29	6.3	14.58±0.55	17.76±5.49
	3.8	15.25±0.59	16.21±3.57	12.5	14.17±0.33	16.20±5.96
	7.5	15.14±0.30	14.52±3.26**	25	14.34±0.59	21.82±17.59
	15	15.24±0.48	12.89±1.18**	50	14.57±0.74	17.22±6.63
	30	15.74±0.47	12.13±0.53**	100	14.22±0.35	21.23±11.54
MHFPK[4]	0	15.44±0.75	18.71±2.49	0	15.38±0.56	18.81±5.44
	18.8	15.63±0.63	19.56±4.19	18.8	15.23±0.38	16.80±4.81
	37.5	16.31±0.88	18.03±3.65	37.5	14.98±0.77	18.10±3.45
	75	16.37±0.83*	17.79±2.72	75	14.74±0.63	18.68±4.92
	150	16.77±0.88*	18.41±2.74	150	14.99±0.41	21.76±7.77
	300	17.12±2.74**	17.66±2.71	300	15.33±0.63	22.79±12.02

[1]Data are mean ± SD with n=7-10. PT is prothrombin time. APTT is activated partial thromboplastin time.

[2]PT – Anova and Dunnett, * = $p < 0.05$, **= $p < 0.01$

[3]APTT – Kruskal-Wallis & Dunnett on Ranks, * = $p < 0.05$, **= $p < 0.01$
[4]For MFHPK all data analyzed as per footnote 2 for male rats and per footnote 2 on log transformed data for female rats (no significant difference of treated from control).

Table S10. Summary of selected hematology data in male SD rats[1,2]

PFAS	Dose (mg/kg-day)	RBC (M/mL)	HGB (g/dL)	HCT (%)	MPV (fL)	ABRETi (10^9/L)	ABNEUT (K/mL)	ABSEOS (K/mL)
PFNAC	0	8.779±0.366	15.12±0.57	48.74±1.85	7.56±0.36	212.03±41.99	5.315±2.495	0.168±0.059
	0.1	8.281±0.895	14.36±1.40	46.10±4.64	7.82±0.50	193.23±33.44	4.163±1.653	0.112±0.047*
	0.3	8.282±0.259*	14.69±0.40	47.33±1.28	7.63±0.44	224.66±50.41	3.679±1.341	0.108±0.037””
	1.0	8.519±0.467**	13.91±0.69**	45.42±2.38**	7.90±0.61	186.29±26.35	2.627±0.787**	0.097±0.026**
	3.0	8.351±0.354	14.09±0.70**	46.18±2.11	7.79±0.43	190.46±33.42	2.351±0.464**	0.095±0.043**
	10	8.118±0.277**	13.84±0.78**	44.76±2.84**	7.66±0.42	172.74±32.27	2.413±0.948**	0.101±0.025**
PFHI	0	8.997±0.329	15.77±0.88	50.40±1.97	7.07±±0.29	231.12±40.52	1.886±0.345	0.159±0.031
	12.5	9.120±0.432	15.92±0.40	51.12±1.63	7.20±0.26	206.20±28.82	1.911±0.373	0.132±0.034
	25	9.149±0.445	15.67±0.55	50.59±1.92	7.13±0.21	198.76±24.13	1.785±0.481	0.136±0.033
	50	9.119±0.347	15.53±0.59	49.78±1.69	7.30±0.36	195.79±17.98*	1.675±0.445	0.160±0.038
	100	9.336±0.374	15.71±0.74	50.60±2.40	7.22±0.38	182.9±23.95**	2.095±0.730	0.147±0.025
	200	8.631±0.367	14.88±0.44*	47.32±1.57**	7.33±0.31	183.6±33.53**	1.692±0.645	0.118±0.030*
CTFPA	0	9.054±0.370	15.70±0.47	50.42±1.52	7.33±0.33	205.26±20.05	1.606±0.212	0.145±0.038
	1.9	9.065±0.402	15.58±0.65	50.28±1.93	7.50±0.37	196.86±16.75	1.988±0.512	0.157±0.041
	3.8	8.648±0.364	15.20±0.71	49.12±2.10	7.79±0.46	209.42±22.32	1.962±0.504	0.152±0.042
	7.5	8.517±0.440*	14.52±0.72**	47.49±2.30*	7.61±0.38	227.78±36.71	2.594±2.472	0.142±0.052
	15	8.356±0.435**	14.62±0.84**	47.72±2.58**	7.56±0.43	210.27±24.45	2.189±0.336**	0.182±0.040
	30	8.306±0.355**	14.44±0.69**	47.32±2.15**	7.54±0.47	192.04±27.08	1.881±0.460	0.145±0.040
MHFPK	0	9.080±0.529	15.85±1.10	51.30±2.95	7.61±0.45	209.12±55.48	2.057±0.917	0.161±0.068
	18.8	8.891±0.355	15.51±0.80	49.80±1.91	7.85±0.72	179.08±30.61	2.172±0.976	0.110±0.032
	37.5	8.732±0.259	15.11±0.46	49.12±1.32	8.18±0.65	192.99±15.30	1.859±0.620	0.179±0.069
	75	9.195±0.417	15.70±0.72	50.90±1.81	7.92±0.76	194.58±21.06	1.731±0.709	0.132±0.036
	150	9.254±0.319	15.30±0.78	50.21±1.86	8.53±0.84*	181.45±26.70	1.761±0.490	0.155±0.028
	300	8.946±0.337	15.18±0.47	49.25±1.52	8.58±0.84*	174.98±22.88	1.674±0.435	0.139±0.055

[1]Data presented as mean ± SD. N =9-10/group for hematology parameters where statistically significant effects were observed for at least one PFAS. RBC – red blood cells, HGB – hemoglobin, HCT – hematocrit, MPV – mean platelet volume, ABRETi – absolute reticulocytes, ABNEUT - absolute neutrophils, ABSEOS =absolute eosinophils

[2] Statistical significance * = $p < 0.05$, ** = $p < 0.01$. Statistical analysis details can be found in study report appendices.

Table S11. Summary of selected hematology data in female SD rats[1,2]

PFAS	Dose (mg/kg-day)	RBC (M/mL)	HGB (g/dL)	HCT (%)	MPV (fL)	ABRETi (10^9/L)	ABNEUT (K/mL)	ABEOS (K/mL)
PFNAC	0	7.097±0.324	13.15±0.57	41.13±1.98	7.61±0.56	173.45±44.02	1.505±0.657	0.086±0.032
	0.3	7.177±0.276	13.21±0.43	41.35±1.64	7.62±0.63	188.23±26.07	1.725±1.311	0.089±0.035
	1.0	7.303±0.562	13.19±0.73	41.13±1.98	7.59±0.74	180.48±14.00	1.468±0.616	0.086±0.028
	3.0	7.262±0.327	13.37±0.68	41.82±2.32	7.77±0.42	176.48±43.69	1.419±0.734	0.083±0.028
	10	7.241±0.358	13.12±0.63	41.08±2.03	7.80±0.60	160.88±34.97	1.562±1.084	0.081±0.022
	30	7.067±0.364	13.04±0.72	40.37±2.37	7.95±0.58	164.87±31.05	1.118±0.358	0.082±0.026
PFHI	0	8.199±0.349	15.33±0.73	48.23±2.20	7.10±0.31	198.18±29.75	1.043±0.170	0.079±0.023
	12.5	8.089±0.401	14.91±0.54	46.90±1.85	7.10±0.30	182.56±33.25	0.936±0.378	0.082±0.026
	25	8.179±0.457	15.38±0.54	48.01±1.96	7.32±0.30	183.09±33.96	1.357±0.634	0.086±0.023
	50	7.963±0.442	15.23±0.72	47.80±2.45	7.47±0.44	208.76±46.02	1.018±0.242	0.079±0.029
	100	7.945±0.270	14.77±0.32	46.45±1.01	7.34±0.51	184.94±29.33	1.058±0.429	0.087±0.029
	200	7.848±0.411	14.58±0.80*	45.73C2.17*	7.68±0.44**	179.34±34.26	0.989±0.168	0.074±0.026
CTFPA	0	8.080±0.428	14.56±0.89	46.42±2.57	7.37±0.43	174.91±25.95	1.125±0.185	0.088±0.020
	6.3	8.284±0.483	15.03±0.80	47.57±2.23	7.57±0.49	181.19±47.49	1.228±0.341	0.100±0.027
	12.5	7.956±0.306	14.52±0.79	45.99±2.31	8.00±0.97	156.62±36.44	1.288±0.550	0.093±0.028
	25	8.081±0.510	14.62±1.04	46.61±2.96	7.65±0.50	166.04±38.39	1.374±0.514	0.113±0.037
	50	7.968±0.317	14.30±0.57	45.75±1.73	8.10±0.46*	177.27±39.00	1.179±0.425	0.117±0.030
	100	8.095±0.472	14.60±0.66	46.76±2.13	8.01±0.50	168.53±20.23	1.208±0.387	0.085±0.025
MHFPK	0	7.920±0.374	14.26±0.68	45.45±2.11	8.01±1.02	185.85±33.32	1.124±0.309	0.084±0.017
	18.8	7.887±0.439	14.58±0.67	46.27±2.37	8.22±0.82	195.85±38.05	1.004±0.296	0.108±0.037
	37.5	7.864±0.307	14.42±0.61	46.13±1.42	8.56±0.67	190.80±35.78	1.068±0.314	0.102±0.040
	75	7.823±0.340	14.40±0.50	45.88±1.35	8.55±0.75	193.71±46.37	1.005±0.441	0.090±0.027
	150	7.731±0.255	14.27±0.54	45.40±1.55	8.84±0.70	193.46±35.08	1.175±0.584	0.093±0.035
	300	8.159±0.351	14.98±0.42*	48.02±1.91*	9.01±0.50*	181.86±36.58	1.220±0.506	0.085±0.022

[1]Data presented as mean ± SD. N =9-10/group for hematology parameters where statistically significant effects were observed for at least one PFAS. RBC – red blood cells, HGB – hemoglobin, HCT – hematocrit, MPV – mean platelet volume, ABRETi – absolute reticulocytes, ABNEUT - absolute neutrophils, absolute eosinophils

[2] Statistical significance * = $p < 0.05$, ** = $p < 0.01$. Statistical analysis details can be found in study report appendices.

Table S12. Summary of selected clinical chemistry data in male SD rats[1,2]

PFAS	Dose(mg/kg-day)	GLUC (mg/dL)	BUN (mg/dL)	CHOL (mg/dL)	ALB (g/dL)	GLOB (g/dL)	A/G (ratio)	ALPi (U/L)
PFNAC	0	93.8±11.3	15.8±2.0	101.7±11.8	3.54±0.18	3.89±0.31	0.91±0.07	127.6±32.8
	0.1	107.6±8.6*	16.2±1.6	73.4±15.5**	3.60±0.21	4.03±0.29	0.90±0.08	138.7±40.9
	0.3	112.1±9.6**	18.3±1.6	78.7±16.8**	3.74±0.23	3.85±0.30	0.98±0.10	174.2±32.7*
	1.0	126.1±10.3**	20.4±2.6**	62.6±7.6**	3.99±0.25**	3.59±0.18*	1.11±0.09	327.4±48.9**
	3.0	126.8±13.8**	22.6±3.6**	68.5±12.2**	4.10±0.25**	3.48±0.13**	1.18±0.10**	286.4±30.8**
	10	122.6±10.8**	22.4±2.6**	68.0±15.7**	3.90±0.20**	3.39±0.19**	1.15±0.10**	315.8±44.0**
PFHI	0	214.5±53.6	16.1±2.2	81.1±16.1	3.38±0.18	3.66±0.24	0.92±0.03	113.6±29.9
	12.5	199.1±29.5	14.8±2.0	87.5±9.2	3.42±0.25	3.77±0.18	0.91±0.04	102.9±14.1
	25	204.5±34.6	15.3±2.1	101.5±15.8	3.35±0.14	3.84±0.36	0.88±0.10	112.3±29.5
	50	196.8±40.7	15.4±1.8	100.2±18.8	3.41±0.12	3.74±0.13	0.91±0.04	103.3±31.6
	100	171.8±39.0	15.7±3.2	112.4±25.0**	3.34±0.53	3.97±0.63	0.87±0.20	103.2±19.1
	200	163.2±18.4*	16.5±2.0	149.1±27.7**	3.55±0.21	3.92±0.23*	0.91±0.04	117.5±29.4
CTFPA	0	189.7±24.7	14.8±1.7	80.5±14.6	3.24±0.16	3.72±0.24	0.87±0.06	120.3±32.8
	1.9	170.1±32.6	14.8±1.8	84.4±9.8	3.35±0.17	3.52±0.20	0.95±0.07	139.8±40.4
	3.8	173.7±32.5	15.6±2.5	79.3±17.3	3.29±0.48	3.65±0.66	0.93±0.20*	131.8±40.1
	7.5	165.6±25.1	15.5±2.2	74.2±8.5	3.39±0.32	3.24±0.19**	1.05±0.14**	159.9±63.1
	15	175.2±15.8	15.8±2.4	63.6±10.8*	3.56±0.21*	3.07±0.19**	1.16±0.11**	237.6±66.2**
	30	175.3±23.2	18.7±2.7**	68.5±10.0	3.72±0.22**	3.14±0.22**	1.19±0.12**	282.9±80.3**
MHFPK	0	198.5±31.6	14.9±1.6	75.3±16.8	3.17±0.19	3.77±0.32	0.85±0.07	115.7±22.5
	18.8	187.8±37.5	14.8±1.4	78.6±12.0	3.17±0.18	3.75±0.18	0.85±0.03	114.0±26.8
	37.5	217.1±33.5	16.3±1.2	75.9±12.3	3.17±0.15	3.69±0.27	0.86±0.07	117.7±20.1
	75	214.9±18.3	15.8±1.8	67.2±7.1	3.18±0.15	3.70±0.19	0.86±0.06	121.9±37.5
	150	206.8±44.7	14.6±1.7	71.1±12.3	3.16±0.18	3.72±0.20	0.85±0.05	123.9±21.1
	300	206.4±24.4	14.8±3.7	76.0±22.0	3.24±0.18	3.70±0.19	0.88±0.07	123.2±28.8

[1]Data presented as mean ± SD. N =9-10/group for clinical chemistry parameters where statistically significant effects were observed more commonly for at least one PFAS. GLUC =glucose, BUN = blood urea nitrogen, CHOL = cholesterol, ALB = albumin, GLOB = globulin, A/G = albumin/globulin ratio, ALPi = alkaline phosphatase.

[2]Statistical significance, * = $p < 0.05$, ** = $p < 0.01$. Statistical analysis details can be found in study report appendices.

Table S13. Summary of selected clinical chemistry data in female SD rats[1,2]

PFAS	Dose(mg/kg-day)	GLUC (mg/dL)	BUN (mg/dL)	CHOL (mg/dL)	ALB (g/dL)	GLOB (g/dL)	A/G (ratio)	ALPi (U/L)
PFNAC	0	98.6±9.6	16.3±2.2	102.4±23.0	4.57±0.33	3.98±0.21	1.15±0.07	63.4±11.5
	0.1	99.4±11.9	15.7±1.3	102.1±16.7	4.36±0.22	3.93±0.12	1.11±0.08	78.0±24.1
	0.3	104.6±13.0	16.8±3.3	99.7±15.0	4.44±0.33	4.05±0.18	1.10±0.06	54.8±10.4
	1.0	100.9±12.7	13.9±2.0	94.3±18.7	4.43±0.26	3.94±0.19	1.13±0.06	52.1±13.7
	3.0	104.4±8.0	16.4±1.3	92.3±16.3	4.56±0.18	3.97±0.13	1.15±0.06	55.4±20.6
	10	108.8±12.0	19.1±3.5	77.7±14.0*	4.66±0.39	3.98±0.29	1.17±0.07	50.5±7.3
PFHI	0	166.9±27.5	16.1±2.9	113.0±19.3	3.99±0.32	3.84±0.11	1.04±0.11	49.8±15.1
	12.5	138.0±29.9*	16.8±2.7	110.5±32.7	4.28±0.37	3.94±0.25	1.09±0.05	42.8±13.0
	25	153.4±30.6	16.2±2.1	114.9±14.1	4.02±0.39	3.78±0.16	1.07±0.11	54.9±21.9
	50	157.1±16.7	15.7±2.2	119.7±25.4	4.27±0.46	3.91±0.26	1.09±0.09	43.7±5.5
	100	153.8±19.2	16.7±2.6	122.7±15.6	4.32±0.36	4.00±0.27	1.08±0.06	49.7±18.4
	200	155.2±21.4	18.0±3.3	154.8±35.9**	4.69±0.33**	4.10±0.33	1.15±0.03*	46.9±15.3
CTFPA	0	144.4±14.4	17.1±3.3	97.5±21.5	4.13±0.46	3.70±0.25	1.12±0.10	57.1±21.5
	1.9	140.7±21.5	16.4±3.6	94.7±23.9	4.07±0.31	3.69±0.19	1.10±0.09	49.7±22.7
	3.8	133.0±25.5	16.5±3.0	95.9±14.5	4.41±0.33	3.78±0.20	1.17±0.10	56.6±19.8
	7.5	143.6±21.3	16.4±2.4	98.2±26.4	4.36±0.42	3.70±0.28	1.18±0.14	54.6±16.8
	15	154.6±21.6	16.3±2.3	96.5±26.2	4.46±0.44	3.73±0.20	1.19±0.08	57.7±20.2
	30	153.4±21.2	18.3±2.5	101.9±15.8	4.62±0.45*	3.77±0.25	1.22±0.07	74.1±17.5
MHFPK	0	155.9±15.3	16.6±3.0	92.2±16.9	4.20±0.36	3.75±0.25	1.12±0.10	61.1±36.6
	18.8	158.0±18.9	15.5±2.2	100.6±17.0	4.26±0.27	3.82±0.36	1.12±0.13	69.1±55.5
	37.5	161.9±28.0	14.7±2.4	106.5±21.4	4.17±0.35	3.99±0.28	1.05±0.08	56.9±24.3
	75	159.0±32.6	16.5±2.1	99.1±21.5	4.45±0.91	3.85±0.96	1.27±0.60	55.0±18.5
	150	166.7±26.3	18.2±2.9	104.3±20.7	4.37±0.51	4.00±0.81	1.14±0.34	47.5±8.8
	300	151.4±28.6	17.6±4.5	107.5±21.4	4.15±0.28	4.16±0.64	1.01±0.13	54.1±22.2

[1]Data presented as mean ± SD. N =9-10/group for clinical chemistry parameters where statistically significant effects were observed more commonly for at least one PFAS. GLUC =glucose, BUN = blood urea nitrogen, CHOL = cholesterol, ALB = albumin, GLOB = globulin, A/G = albumin/globulin ratio, ALPi = alkaline phosphatase.

[2] Statistical significance * = $p < 0.05$, ** = $p < 0.01$. Statistical analysis details can be found in study report appendices.
